# Immunity: defense against infections essential for all living organisms

**DOI:** 10.3389/fimmu.2026.1840774

**Published:** 2026-06-23

**Authors:** Srinivas Akula, Sara Wernersson, Lars Hellman

**Affiliations:** 1 Department of Cell and Molecular Biology, Uppsala University, The Biomedical Center, Uppsala, Sweden; 2 Department of Animal Biosciences, Swedish University of Agricultural Sciences, Uppsala, Sweden

**Keywords:** adaptive immunity, CRISPR-Cas, evolution, Fc-receptors, immunoglobulins, innate immunity, MHC, restriction enzymes

## Abstract

All organisms need protection against infection. Bacteria are often primarily seen as infectious agents, but they also need protection against bacterial viruses, so-called bacteriophages. To this end, bacteria have developed very complex defense systems, including apoptosis-like mechanisms, restriction enzymes, and even adaptive-type mechanisms involving immunological memory of immune responses through a system called CRISPR–Cas. An earlier dominating view was that adaptive immunity in eukaryotes only exists in jawed vertebrates, as their immune system includes the classical and highly variable immunoglobulins (Igs) and T-cell receptors (TCR). However, other types of variable molecules, which may be involved in immunity, have also been identified in insects, snails, lancelets, plants, sea urchins, and jawless fishes. Interestingly, fishes without jaws, such as the hagfish and lamprey, have a very complex adaptive immunity built on lymphocyte-like cells and variable lymphocyte receptors (VLRs). Notably, the variability of these VLRs has been estimated to be in the same range as Igs and T-cell receptors. This illustrates that very diverse strategies have been used to create an adaptive immune system in different organisms, indicating potent convergent evolution. Vertebrate immunity includes both adaptive and non-adaptive components, which work closely together to form a very powerful immune system for defense against infections. In contrast to adaptive immunity, the majority of the non-adaptive innate defense mechanisms, such as pattern recognition receptors, antimicrobial peptides (AMPs), iron-binding proteins, the complement system, and lysozymes, can be traced back to early eukaryotes. Immunity of invertebrates seems to rely almost entirely on innate defense mechanisms, while the presence of complex adaptive mechanisms in invertebrates, such as the VLRs of jawless fishes and Igs and TCR of jawed vertebrates, is questionable. This review summarizes old and recent findings of importance for our understanding of how immunity became an integrated part of all living organisms, from bacteria to humans, and the very different strategies that different organisms use in the protection against infection.

## 1 Introduction

Both adaptive and non-adaptive, so-called innate mechanisms participate in the defense against infection in both bacteria and eukaryotes. Innate mechanisms have developed specificity over millions of years by targeting molecules that are found in different infectious organisms, but not in self, but is not adapted in the organism upon infection, whereas the specificity of the adaptive systems in general develops in the infected organisms or, as for the bacteria, in the present population of cells. In bacteria, several innate mechanisms have been identified, including restriction enzymes that cleave and inactivate incoming DNA (1). Bacteria also have an apoptosis-like mechanism where the infected cell kills itself for the benefit of the population by producing toxins (2). Adaptive immunity in both Eubacteria and Archaea is provided by the CRISPR–Cas system, where the bacteria remember phages that the population has encountered (3). Short 20–40 nucleotide regions from the phage genome are saved in a cassette to be transcribed and used as guiding RNA to target these sequences in incoming DNA for subsequent degradation.

In almost all multicellular eukaryotes, we find several types of innate immune mechanisms, including sensors of foreign structures, so-called pattern recognition receptors (PRRs), as well as antimicrobial peptides (AMPs), iron-binding proteins, the complement system, lysozymes, and several immune cells. Some of these immune cells have the capacity to engulf incoming pathogens by phagocytosis. The PRRs can bind bacterial, fungal, and parasite-specific structures, so-called pathogen-associated molecular patterns (PAMPs), and thereby activate the immune system by producing signaling molecules, including cytokines, chemokines, and interferons, to make the body aware of the presence of an invader and attract other inflammatory cells to the site of infection (4). Lysozyme, which is present in the circulation, at mucosal surfaces, in saliva, and in tears, is produced by epithelial cells or phagocytic cells, cleaves a specific bond between two sugar units in the bacterial cell wall, and thereby strips bacteria of their cell wall. The removal of the protective cell wall interferes with bacterial cell division and makes the bacteria much more sensitive to osmotic lysis and membrane-targeting antibacterial peptides. Following recognition and inactivation, the pathogen can be taken care of by the phagocytic cells, which engulf incoming microbes and kill them inside the cell by various mechanisms, including oxygen radicals, nitric oxide (NO), and an array of enzymes of the lysosomes. Together, these enzymes and highly reactive low molecular weight compounds can inactivate almost any macromolecule. Most of these innate immune mechanisms, including PRRs, lysozymes, and phagocytic cells, are old and found in most multicellular organisms.

Adaptive immunity is also found in many multicellular organisms. The most detailed picture of the adaptive defense systems has been obtained for mammals, which encompass intricate variability-generating mechanisms producing enormous numbers (estimated to 10^14^) of different immunoglobulins (Igs) (also called antibodies). These Igs are generated against specific structures on or connected to an incoming pathogen, thus allowing for highly specific recognition and targeting of intruders. The mammalian adaptive immunity also includes highly variable T-cell receptors (TCRs). These TCRs are involved in the selection and expansion of cytotoxic T cells to target host cells that are infected with intracellular pathogens, such as viruses and intracellular bacteria, or to target modified (mutated) self-cells in tumors.

Here, we provide an overview of different protective mechanisms used by different organisms to show the complexity of strategies used in immunity to infection across different organisms.

## 2 Immunity in bacteria

**Figure 1:**
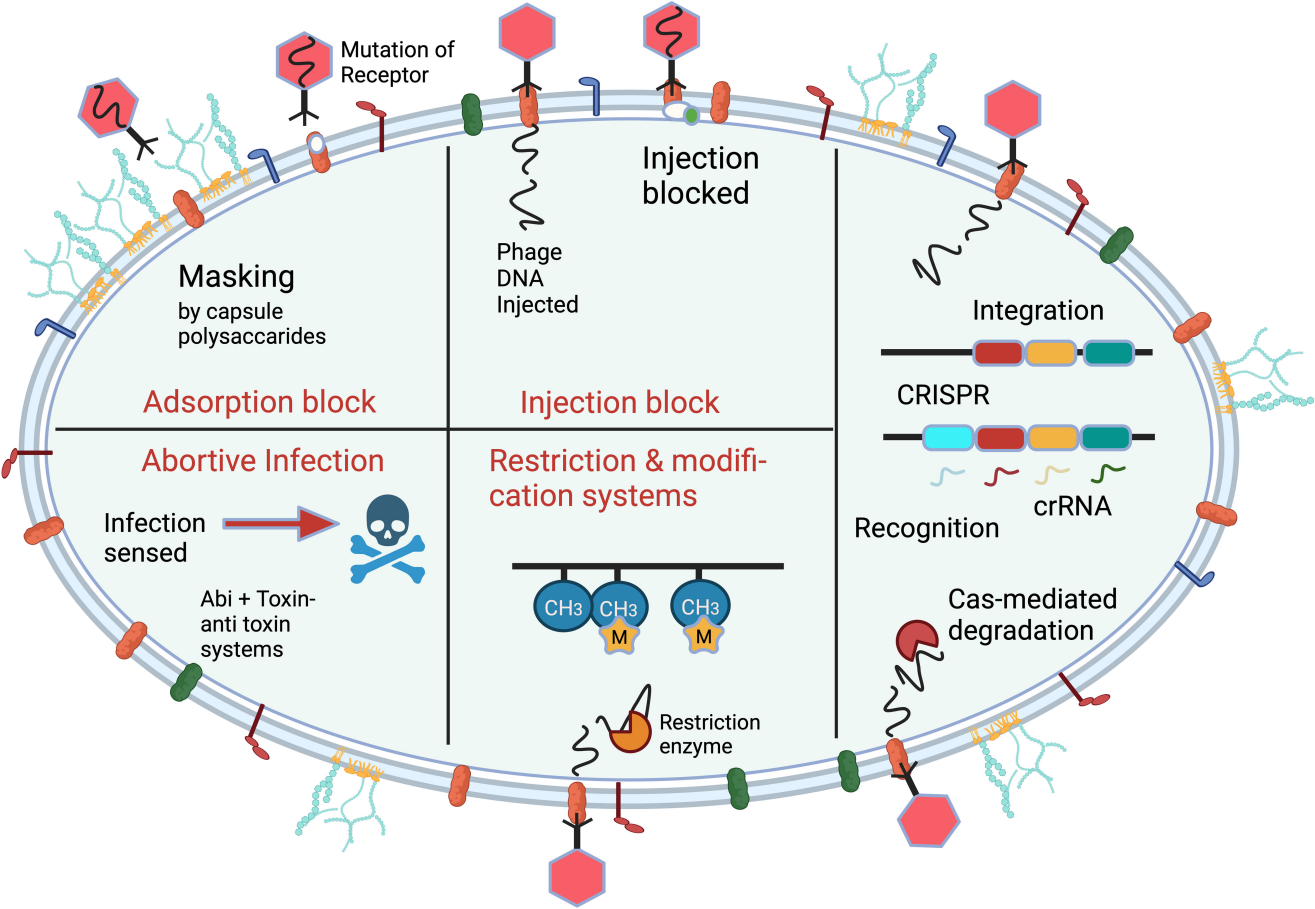
Bacterial defense systems against phages (modified from Westra et al., *Ann. Rev. Gen*, 2012) (6). A summary of different defense mechanisms used by bacteria to defend themselves from infections by bacteriophages, including masking, mutations of receptors, blocking of phage DNA injection, restriction enzymes to cleave incoming DNA, abortive infection, and the CRISPR–Cas system. The figure is made in BioRender.

Bacteria and Archaea are both under constant threat from massive numbers of phages. Seawater has been found to contain millions of phages per milliliter, and the number exceeds that of bacteria and Archaea by approximately 15-fold (5). Estimates indicate that phages may remove 20%–40% of the prokaryotes in the upper levels of the seawater by lysis every day (5). Bacteria, therefore, need efficient ways to protect themselves from infection. The defense systems that have been identified in bacteria include mechanisms for inhibiting the entry of the phages, such as masking by capsule polysaccharides and mutations of the receptor to avoid binding (**Figure 1**) (6). If the phage manages to pass these initial defense layers and manages to inject its DNA into the bacteria, the bacteria will use several different mechanisms to avoid being killed or to prevent spreading the phage in the population. Restriction enzymes cleave and thereby inactivate the infectious DNA. Cell death or metabolic arrest can be induced in the host before the phage has had time to multiply by the strategy of abortive infection (Abi) (**Figure 1**). This latter mechanism acts similarly to apoptosis induction in mammals to protect the cell population by inhibiting virus propagation and spread (2). This can be described as an altruistic mechanism to protect the population by sacrificing the individual (7).

### 2.1 Restriction and modifying systems

The restriction and modifying systems are by far the most widespread defense systems and are present in more than 90% of all bacterial and archaeal genomes (1). There are four different types of restriction enzymes. Types I, II, and III are classical restriction enzymes recognizing 4–8 nt regions and then cutting at non-methylated sites within the recognition sequence or at a certain distance away from the site. Type IV enzymes recognize instead modified bases that are found in phage DNA but not in host DNA. Types I, II, and III, but not Type IV, depend on methylases to protect self-DNA (1). Phages may methylate their DNA by their own methyltransferases or acquire such from the bacterial host to protect their DNA from degradation, modify bases in their DNA using uridine or glycosylated 5-hydroxymethylcytosine, or have proteins that block restriction enzymes (8–11). Multiple restriction and modifying systems, therefore, most likely exist to counteract the evasion of phages to become degraded by these systems as a part of a constant arms race.

### 2.2 Ssp-systems

Some bacteria lack the methyl transferases needed for protection against enzyme cleavage, and these bacteria often have an alternative protective system using phosphorothioation to protect self-DNA from degradation (12, 13). These systems have been named Ssp systems and are often present in bacteria that lack a restriction and modification system entirely, such as described in the previous section (12, 13). These systems insert a sulfur atom in the linker between bases in the DNA as a double-stranded phosphodiester modification (12). These modification enzymes are accompanied by other components of the system that can degrade unmodified phage DNA, similar to the classical restriction modification systems (12).

### 2.3 The Argonaute system

Both Archaea and Eubacteria use the Argonaute system to protect the host against genetically mobile elements, including phages, plasmids, and transposons (14). It is a system that uses short RNA or DNA sequences to target incoming RNA or DNA, similar to the CRISPR–Cas system. However, the short RNA or DNA pieces are not encoded within the bacterial genome but generated by still unknown mechanisms from RNA or non-chromosomal DNA (14). The Argonaute proteins confer immunity in all domains of life, and the mechanisms of generating the short RNA or DNA sequences for targeting are better characterized in eukaryotes, where two RNase III variants trim double-stranded RNA into the short sequences used by the eukaryotic Argos (eAgos): first, Drosha in the nucleus and then Dicer in the cytoplasm (14). No direct homologs to Drosha and Dicer seem to have been found in bacteria or Archaea (14). Recently, three types of short pArgo systems have been identified in different bacterial strains, which use different structural domains and short RNA guides to identify foreign nucleic acids: the SPARSA, SPARTA, and SPARDA systems. The SPARSA uses SIR2 domains, the SPARTA uses TIR domains, and the SPARDA uses a nuclease domain (15–17). The first two systems form active tetramers with NADase activity that deplete cellular NAD+, which results in cell death. The tetramers of the SPARDA system do instead activate the nuclease to degrade both foreign nucleic acids and genomic DNA as well as cellular RNA (17).

### 2.4 Abortive infection systems

Abi systems are also commonly used resistance strategies against phage infections. In these systems, the infected cells are either committing suicide before the phage can complete its life cycle or slowing down their own metabolism to give other systems, such as restriction enzymes or CRISPR–Cas, the chance to inactivate the phages (2). The Abi systems can sense the presence of phages by sensing intermediates of phage replication, structural phage proteins, extensive phage transcription, or shutdown of host transcription (2, 18). The most well-characterized Abi systems are the Rex, the PifA, and the Lit and PrrC of *Escherichia coli*, and the abiZ system of *Lactococcus lactis*. The Rex system acts on several lambdoid phages and particular strains of the T4, T7, and T5 phages (2).

Recently, a new family of Abi systems has also been described. In these systems, which collectively are called CBASS, the sensing and the killing module do not physically interact but signal through a small secondary messenger (3). These secondary messengers consist of two or three nucleotides covalently linked, forming a cyclic molecule (3). In the most well-characterized example, a protein called DncV produces cyclic GMP-AMP (cGAMP) upon activation, which leads to the activation of a phospholipase that degrades the inner membrane of the bacteria, leading to cell lysis and death (3).

### 2.5 Toxin–antitoxin systems

A few of the Abi systems in bacteria belong to the toxin–antitoxin systems, which are found in most bacterial and archaeal genomes, and individual species often encode many distinct systems. The toxin–antitoxin systems regulate cell growth and survival by encoding a stable toxin and an unstable cognate antitoxin. The antitoxin can, under certain conditions such as stress, be degraded, which results in the toxin inducing growth arrest or programmed cell death. Some bacterial genomes encode as many as 80 such systems (3). Interestingly, despite their abundance and prevalence, the functions of these toxin–antitoxin systems are still poorly defined and even controversial (18). They are also not highly conserved even among closely related bacterial strains (18). They have been indicated to play a role in the stress response, conferring resistance to antibiotics via persistence, in the regulation of biofilm formation, in plasmid maintenance, and in phage resistance via Abi (2, 18, 19). However, their role in stress and antibiotic resistance has been questioned due to minor effects when the systems have been deleted (18). Their role in Abi is still also limited to relatively few well-established examples, such as the RnlAB in *E. coli* K12 and the ToxIN system in *Erwinia*, *Serratia*, and *E. coli* (2, 18). However, more evidence from a few additional systems now seems to favor these systems as primarily involved in immunity to phages (7, 18). It has also been argued that the high variability of the toxin–antitoxin systems is an effect of a frequent and rapid diversification of the system and thereby a way for bacteria to stay one step ahead of their predators (18). The strongest evidence for the role of toxin–antitoxin systems in phage defense is coming from the phage strategies to counteract these systems (18). At least three mechanisms to overcome the toxin–antitoxin systems have been identified, including preventing the activation of the system, modifying the toxin or the target, and producing antitoxins or antitoxin mimics (18). Interestingly, the fact that many toxin–antitoxin systems are found on prophages or genomic regions containing phage-related genes indicates that they are used to protect the bacterial cells from superinfection with other phages (18).

### 2.6 CRISPR–Cas systems

In addition to the above innate type mechanisms, bacteria have a type of adaptive immune system with similarities to the mammalian adaptive immune system by having memory of previous encounters with infectious organisms, the clustered regularly interspaced short palindromic repeats (CRISPR–Cas). CRISPR–Cas systems are found in both bacteria and Archaea (**Figure 1**). In some cases, the CRISPR–Cas systems, particularly Type III, can also be involved in Abi (3). These CRISPR–Cas systems represent a form of adaptive immunity by copying a 20–40 nt region of the phage DNA and inserting these sequences into an expression cassette in the bacterial chromosome (20). This cassette contains several such sequences from phages that the bacterial population has encountered. Upon transcription, the resulting small RNA pieces are trimmed to the desired size by nucleases and bound to the CRISPR protein complex to be used for screening DNA that enters the cell. If the protein RNA complex recognizes that sequence in the DNA within the cell, the Cas nucleases are recruited. The Cas nuclease then starts degrading the DNA and thereby inactivates the intruder. Interestingly, this system has its flaws similar to mammalian adaptive immunity; the bacteria may become autoimmune. Despite mechanisms to limit self-DNA integration in the CRISPR-cassettes, an analysis of a large panel of prokaryotic genomes shows that approximately 0.4% of all spacer sequences are self-sequences and that approximately 18% of all genomes analyzed contained CRISPR-cassettes with at least one self-targeting sequence (21, 22). This indicates that the system potentially can recognize the chromosomal DNA for destruction, which results in the death of the bacteria by an autoimmune mechanism (23). To what extent this actually happens is, however, difficult to estimate, and additional mechanisms may be involved in limiting an autoimmune attack on self-DNA (24).

The CRISPR–Cas system has only been found in approximately 30%–40% of Eubacteria but in more than 90% of Archaea, indicating that many bacterial and some archaeal species can survive without an adaptive immune system (3). There are also relatively large variations in the number of components and how they are organized within these CRISPR–Cas systems. Currently, two classes, seven types, and 46 subtypes of CRISPR–Cas systems have been identified (25). Type I and type III systems together represent 90% of all detected CRISPR–Cas loci in Eubacteria and 100% in Archaea (26). Type I and Type II systems both degrade phage DNA, thereby avoiding cell death, whereas Type III sometimes leads to Abi, and thereby cell death (3). Type III may act as a rescue system if the infection has gone beyond a stage where the cell can be saved (2). The evolution of the CRISPR–Cas systems is also very complex, involving transposons, elements from toxin–antitoxin systems, gene duplications, and numerous rearrangements (26). Some of these systems are especially prone to shuffle the adaptation module and effector modules from different CRISPR–Cas subtypes, most likely as a consequence of an arms race against rapidly evolving viruses (26). Type II includes Cas9, a multi-subunit protein with several enzyme activities, which is now commonly used as a tool in genome editing (27, 28). Cas9 includes both recognition and nuclease in the same polypeptide and is therefore easier to use than the other multi-subunit systems (27).

All of these immune mechanisms come with a cost. A stringent system can also inhibit DNA from other bacteria from entering the cell and thereby limit the exchange of genetic information between bacterial cells by conjugation. Such a limitation may affect the adaptability of the bacterial population to a changing environment. A less stringent system of immunity within a bacterial population can therefore be advantageous (29).

Phages also develop ways to avoid being degraded by the restriction enzymes by reducing the number of cleavage sites and modifying bases to block cleavage. Anti-CRISPR–Cas and anti-Abi systems have also been detected, indicating that there is a constant ongoing arms race between host and parasite. Key factors for bacteria, as for other organisms, most likely include the cost, the effectiveness, and the potency to adapt the system for new challenges (2, 18, 29). Bacteria often share immune genes by horizontal transfer, and phages can carry genes that are parts of these protective systems to become incorporated in the genome of the host as part of a competitive strategy against other phages (13). Individual cells within a bacterial population can also have major differences in which anti-viral protective systems they carry (13).

Studies of the different systems to protect bacteria and Archaea from phage infections have shown an extreme complexity and adaptability of these systems, and these systems are not lagging in complexity compared to immune mechanisms in complex multicellular organisms.

The evolution of an adaptive immune system, such as the CRISPR–Cas in bacteria and Archaea, has most likely been the product of a potent arms race between phages and bacteria, similar to what we later in this review will see where highly complex adaptive immune systems have developed in parallel in jawless and jawed vertebrates.

## 3 Innate immunity in multicellular eukaryotes

A number of different mechanisms participate in generating a powerful innate immune system in multicellular organisms. The majority of these mechanisms, including antimicrobial peptides, lysozymes, iron-binding proteins, the complement system, and PRRs, seem to be very old.

### 3.1 Pattern recognition receptors

The PRRs are there to detect the presence of common structures of pathogens. The function of these receptors is to sense if pathogens have entered our tissues and to alert the immune system. These systems involve Toll-like receptors (TLRs), NOD-like receptors (NLRs), RIG-I-like receptors (RLRs), and a large number of additional receptors, as well as the signaling pathways and transcription factors to induce a response to non-self structures. One of the most conserved and evolutionarily oldest of these transcription factors is the nuclear factor kappa B (NF-κB). NF-κB has been found in all multicellular organisms, from sponges to humans (30, 31). NF-κB-like proteins have also been found in some single-celled organisms such as the holozoan *Capsaspora owczarzaki*, indicating a very early appearance (30, 31). However, this signaling pathway has also been lost in some species, such as in the nematode *Caenorhabditis elegans* (30, 31). NF-κB belongs to a gene family of related molecules, that is, the Rel family, which is also involved in immune signaling. The Rel family members seem to have been added somewhat later than NF-κB during multicellular evolution and have so far only been found in *Arthropoda*, *Mollusca*, and *Chordata*, but not in Cnidaria and sponges (30).

**Figure 2:**
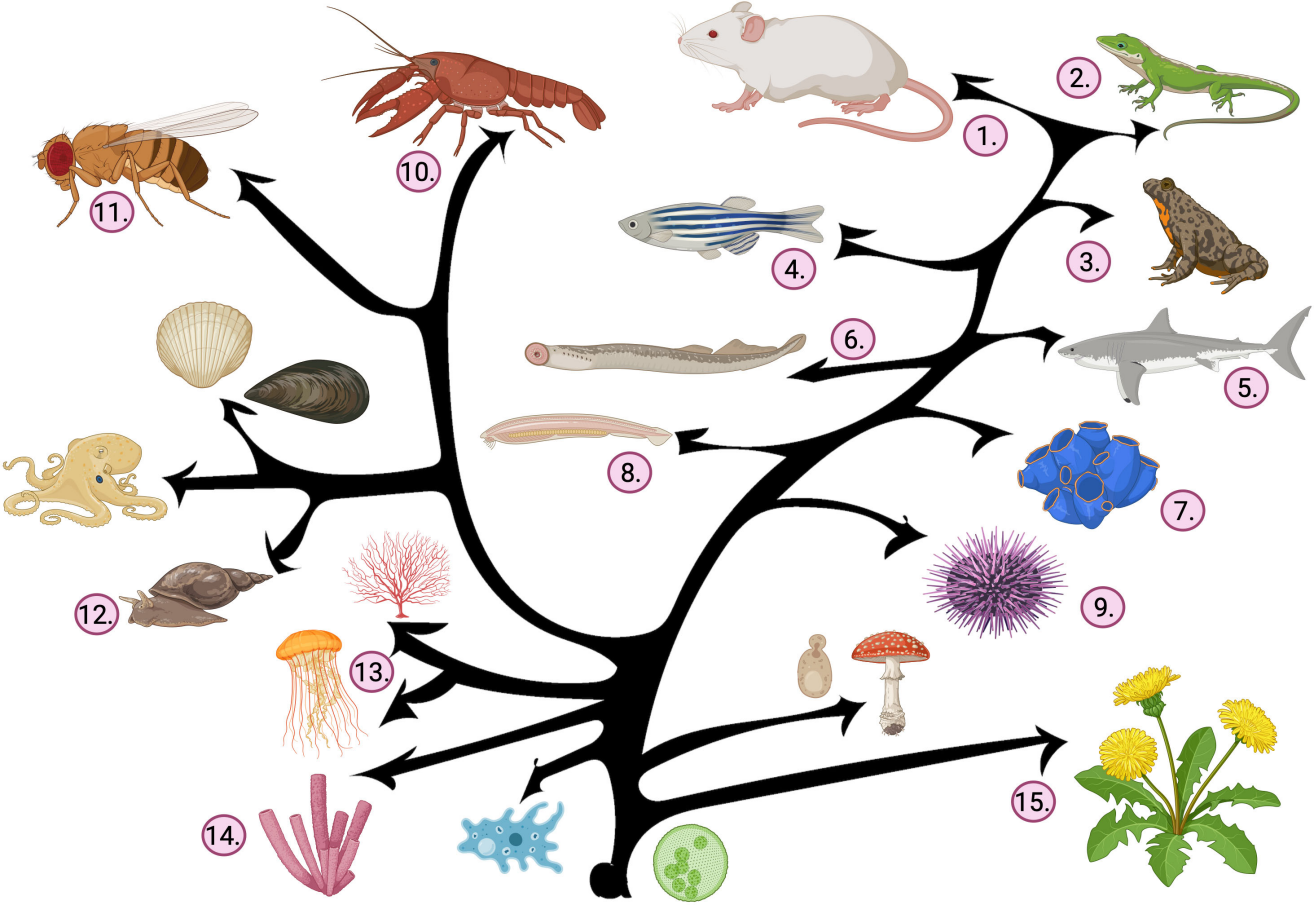
A schematic figure of the evolution of multicellular organisms. 1) Mammals, 2) reptiles, 3) amphibians, 4) bony fishes, 5) cartilaginous fishes, 6) jawless fishes, 7) tunicates, 8) lancelets, 9) echinoderms, 10) crustaceans, 11) insects, 12) gastropods, 13) cnidarians, 14) sponges, and 15) plants. The figure is made in BioRender. The organisms at the base of the tree are the ones that appear first during evolution. Single-celled algae already 1.5–2 billion years ago; land plants 470 million years ago, first as moss-like plants and later, approximately 410 million years ago, as plants with real roots and vascular system. Sponges and Cnidaria most likely originated approximately 700–800 million years ago. The first chordates, echinoderms, molluscs, crustaceans, and insect ancestors seem to have appeared during the early Cambrian, 543–510 million years ago. Sharks approximately 450 million years ago, amphibians approximately 370 million years ago, and placental mammals most likely approximately 120 million years ago. It is sometimes difficult to provide exact time estimates because there are relatively large discrepancies between fossil record and the timing based on the molecular clock.

To be able to follow the different animal groups and their appearance during evolution, we include a simplified picture of the evolution of multicellular organisms (**Figure 2**).

**Figure 3:**
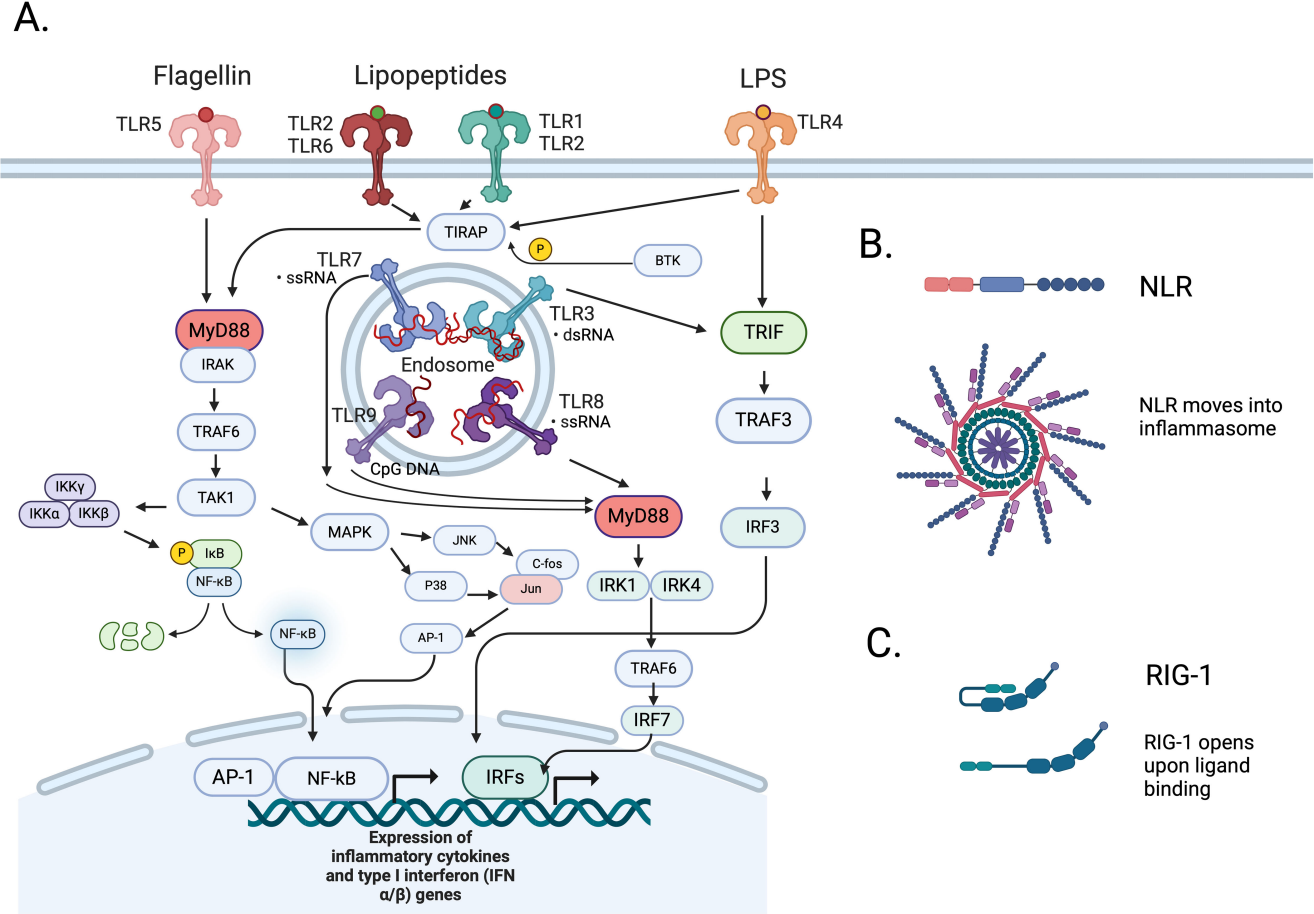
Pattern recognition receptors. Modified from Chen et al., 2024 (4). A large number of pattern recognition receptors have been identified in mammals, and many of them are found in almost all multicellular organisms. Three of the most well-characterized are the Toll-like receptors (TLRs), the NOD-like receptors (NLRs), and RIG-1. They recognize several conserved structures found in many pathogens, such as lipopolysaccharides from Gram-negative bacteria, lipopeptides, flagellin components, cell-wall components of bacteria and fungi, double-stranded RNA, non-methylated DNA, and many additional conserved compounds of pathogens. The figure also shows several of the signaling pathways involved in transferring the information from the PRRs to the nuclei and thereby starting the production of effector molecules in the inflammatory response. **(A)** shows some of the different human TLRs and the signaling events involved in their activation of response genes. **(B)** shows a schematic structure of an NOD-like receptor and how it is integrated into the large cytoplasmic inflammasome. **(C)** shows a schematic structure of the RIG receptor and how it changes structure upon ligand binding. The figure is made in BioRender.

The PRRs are essential for a rapid response to incoming pathogens by activating phagocytic cells and inducing the production of inflammatory cytokines and potent chemotactic substances such as chemokines and several arachidonic acid metabolites (**Figure 3**) (4). In the human genome, we have 10 members of the TLR family, which bind different non-self molecules (4). These non-self molecules are characteristic structures of various pathogens, such as bacterial lipopolysaccharide (LPS) from *E. coli*, which bind to and activate TLR4 (**Figure 3**). Similarly, double-stranded RNA from viruses binds TLR3, and non-methylated CpG-containing DNA binds TLR9 (**Figure 3**) (4). For some immune cells, this response is very fast and extremely potent, such as for human monocytes. As an example, within 4 hours of stimulation with the bacterial LPS from the outer membrane of *E. coli*, human blood monocytes very strongly upregulated a limited number of inflammatory cytokines and chemokines (32). Notably, LPS only induced the expression of four of the more than 40 cytokines at significant levels; the four classical inflammatory cytokines, IL-1α, IL-1β, IL-6, TNF-α, and IL-8 (a chemokine), became the most highly expressed proteins in the cell after a 4-hour incubation with LPS (32). Interestingly, the response to this non-self molecule was both very rapid and very potent, even if the mRNA expression levels of the different TLR genes were very low (33).

TLRs most likely first appeared in cnidarians, as they are found in some Cnidaria species but seem to be absent in Porifera (sponges). However, some cnidarian species lack TLRs, and TLRs have most likely also been lost in Bryozoa and Platyhelminthes (34). The number of members also differs markedly between different species, with 10 in humans, only one in the roundworm *C. elegans*, and as many as 280 in one mussel species, *Mytilus edulis* (34).

The founding member of the TLR family, TLR, was first identified in the fruit fly *Drosophila melanogaster*, where it was involved in forming the dorso-ventral polarity in the developing embryo, and it was only later that it was found to be essential for antifungal immunity (35). *Drosophila* has nine Toll family members, which are primarily involved in pathogen detection and also in tissue development and tissue homeostasis (35).

### 3.2 The cGAS–STING system

Relatively recently, a new sensing system to detect foreign DNA in the cytoplasm has been identified, the cyclic GMP–AMP synthase system (36). This system detects cytosolic DNA as a sign of infection or cellular damage and induces a type I interferon and an inflammatory response (36). The cGAS senses cytoplasmic DNA and produces 2′3′-cyclic GMP-AMP, which activates the stimulator of interferon genes (STING), which, in turn, activates TANK-binding kinase 1 (TBK1) and interferon regulatory factor (IRF3), which induce interferon production. The activation of STING also results in the activation of NF-κB and an inflammatory response (36). This system detects viral DNA and intracellular bacteria. However, the accumulation of cellular DNA can also result in the misregulation of the system and the induction of autoimmunity, such as systemic lupus erythematosus (SLE) (36).

### 3.3 Lysozyme

Lysozyme is an enzyme that strips bacterial cells of their protective outer layer, the carbohydrate-containing cell wall. Having a damaged cell wall makes the bacteria more sensitive to immune attack. Lysozyme is present in the circulation, at mucosal surfaces, in saliva, and in tears and is produced by epithelial cells or phagocytic cells. The major lysozyme that in mammals is involved in bacterial defense is the chicken type, lysozyme C (37, 38). However, there are several additional members of the large lysozyme multigene family. The different members of this family are important components of the innate immune system by cleaving a specific bond between two sugar units in the bacterial cell wall, which destabilizes the cell wall and finally partly or completely removes it, making the cell sensitive to AMPs and osmotic changes. The different lysozymes are therefore an important factor in the defense against bacteria, primarily Gram-positive bacteria. Gram-positive bacteria have their cell wall exposed due to the lack of an outer membrane that is present in Gram-negative bacteria. Lysozyme C, which is found in all vertebrates, is an important component in the bacterial killing capacity of several human immune cells, including monocytes, macrophages, and neutrophils. Lysozyme C is actually one of the most highly expressed transcripts in human blood monocytes (33). Other family members, such as goose-type lysozyme G, invertebrate-type lysozyme I, and the less common *Chalaropsis* type, are found in other animal species, including many invertebrate species, where lysozymes have both digestive and immune functions (39). Lysozymes are also found in bacteriophages (phage type), bacteria (bacterial-type), and in plants (plant-type) (39). Lysozyme G is also found in mammals but has not been found to have a role in immunity (39). Lysozyme G is found in myxobacteria, where it has important digestive functions, and these lysozyme genes show relatively high sequence similarity with animal lysozyme G;s, indicating that animal lysozyme G;s may originate from horizontal transfer of genes (39). Lysozyme G also plays both digestive and immune functions in gastropods (39). The human lysozyme G genes are not expressed at significant levels in five analyzed immune cells but seem instead to be expressed in the kidney and liver (33, 39). Despite very limited sequence similarity between these different lysozyme types, they exhibit conserved protein 3D structures and conserved enzyme mechanisms, indicating convergent evolution across eukaryotic kingdoms from plants to animals and even prokaryotes (39, 40).

### 3.4 Metal ion-binding proteins

An additional strategy to limit the growth and spread of pathogenic bacteria is to limit access to specific metal ions needed for their propagation. One essential such component is iron, which is needed for several, often redox, enzymes. In mammals, lactoferrin is a protein of major importance for limiting access of iron for bacteria in infected areas (41). Lactoferrin is found in high concentrations in milk, in particular, the early milk of the colostrum (41). It is also found at high levels in the secondary granules of neutrophils and at mucosal surfaces in the respiratory, urinary, reproductive, and intestinal tracts (41). Lactoferrin binds iron with extremely high affinity, and this binding is also very resistant to low pH (41). Lactoferrin binds Fe^3+^ with a 300 times higher affinity than another human iron-binding protein, transferrin (41). A highly positively charged N-terminal fragment of lactoferrin can also be released from the N-terminal of lactoferrin by proteolytic cleavage and acts as an antibacterial peptide, named lactoferricin (41). This fragment and the full-size lactoferrin have, due to their positive charge, been indicated to act as negative regulators of excessive inflammatory reactions. This is achieved by their ability to bind to the inflammatory mediators LPS, LPS-binding proteins such as CD14, and free DNA, thereby limiting their access to PRRs (41). The importance of iron for bacteria is also shown by the presence of counteracting proteins from the pathogenic bacteria to try to outcompete lactoferrin for available iron. Such iron-binding proteins produced by almost all Eubacteria are named siderophores (42). Their presence in Archaea has not been studied in detail; however, relatively recently, a few siderophore genes in *Archaea* were described, which seem to originate from Eubacteria by horizontal transfer (43). Interestingly, studies have indicated that archaeal species can use siderophores produced by Eubacteria to obtain iron ions when living in mixed communities and that the secretion and uptake of siderophores have a major ecological impact on bacterial communities (42, 43). Lactoferrin is also expressed in the afferent tubules of the human kidney, indicating a role in the reabsorption of iron from the urine and possibly also in limiting potential bacterial growth in urine (44).

### 3.5 Antimicrobial peptides

AMPs are an essential part of the innate immune system in multicellular eukaryotes. They constitute a large number of related and unrelated, often relatively short peptides/proteins, where the majority are cationic and have amphipathic characteristics (45). However, negatively charged AMPs also exist (45). Amphipathic means that they have one side that is charged, most often positively charged with arginines and lysines, and that the other side is hydrophobic; i.e., it does not like water. The hydrophobic side often contains aromatic and aliphatic amino acids. These antimicrobial peptides have the capacity to bind to bacterial and parasite membranes due to the presence of negatively charged phospholipids such as phosphatidylserine in the outer layer of their membranes. Most human cells, probably also mammalian and possibly also the majority of vertebrate cells, have asymmetric membranes with low levels of a negative charge in the outer layer of their cell membranes; because phosphatidylserine is actively shunted to the inner side of the membrane by the flippase, such cells are less sensitive to these peptides (46, 47). After binding to the cell membrane of the bacteria, the hydrophobic part of the peptide tries to enter the inner side of the lipid bilayer to avoid the water environment. When present in large numbers, these peptides form pores in the bacterial membrane, thereby facilitating the entry of calcium and sodium ions into the cell and thus depolarizing the membrane. The influx of calcium results in the activation of apoptosis-like mechanisms, and the depolarization of the membrane results in the uncoupling of the energy-generating mechanisms of the bacterial cell and subsequently in the death of the cell. Other models for the action of these peptides have also been proposed, including general destabilization of the membrane by forming large aggregates on the cell surface and also numerous other antimicrobial actions that are not only membrane-dependent, such as targeting ribosomes, inhibiting proteases, inhibiting nucleic acid biosynthesis, and inhibiting cell division (45).

Such antimicrobial peptides seem to be present in almost all multicellular organisms and also in many unicellular organisms (48). They are actually also relatively common in both Gram-positive and Gram-negative bacteria and in fungi, where a majority of them target membranes and form pores; numerous other activities have also been described (48). These peptides represent a very diverse family of different structures with varying degrees of antimicrobial activity, some with broad spectrum, whereas others have relatively narrow specificity to only a few bacterial types. They are very diverse in sequence and small in size, generally between 10 and 100 amino acids, which has made it difficult to purify them in sufficient quantities for a more detailed analysis. However, in some species, a more detailed picture of their presence has been obtained. Amphibians have the largest number of described AMPs, with 1, 117 entries in a large AMP database (2020) and with 14 different types, including bombinins, buforins, cathelicidins, dermaseptins, esculentins, fallaxin, magainins, maximins, phylloceptins, phylloxins, plasticins, plasturins, pseudins, and ranatuerins (48). These peptides have been found to have antibacterial, antifungal, anti-leishmanicidal, and anti-tumor activities (48). AMPs are very important in insect immunity, and a number of such peptides have been isolated and characterized in the fruit fly *D. melanogaster*, including cecropins, defensins, drosomycins, drosocins, attacins, and metchnikowins (49). In mammals, some of these peptides are constitutively expressed and are important components of the granule content of inflammatory cells, such as neutrophils, whereas others are induced upon infection, primarily at mucosal surfaces and in the skin. Antibacterial peptides are also present in plants, which further shows their broad use in almost all multicellular organisms (50). Interestingly, in plants, these peptides are almost exclusively cysteine-bridged for stabilization. All from one to six cysteine bridges have been observed (50). Plants have a large number of different AMPs, including thionins, defensins, hevein-like peptides, knottin-type peptides, α-hairpinins, lipid transfer proteins, snakins, and non-cysteine-rich peptides (48). The majority of them have both antibacterial and antifungal activities; however, several of them also have anti-insecticidal or anti-tumor activities (50). These plant peptides also have additional functions beyond being membrane active, such as significantly affecting plant growth and development (50). Most of these plant AMPs seem to be expressed constitutively at basal levels but are also rapidly transcribed and thereby markedly increased in amount after being induced by pathogens (50).

### 3.6 The complement system

In mammals, the complement system is a complex system of more than 30 different soluble and membrane-bound components with three different ways of activation: the classical, which is dependent on the complement component C1q and on antibodies (IgM and IgG); the lectin pathway, which is dependent on a mannose-binding lectin (MBL) and ficolins; and finally, the alternative pathway, which is dependent on a low degree of self-activation by an unstable thioester bond in the central C3 component (51). Some of the components are found in almost the entire animal kingdom, but the full set of components, as we see in humans, first appeared in jawed vertebrates. The first component of the complement system to appear during evolution seems to be C3, which has been found already in cnidarians, but not in sponges, indicating that it appeared in an early cnidarian ancestor more than 500 million years ago (52). C3 belongs to a larger family of thioester-containing proteins, including α_2_-macroglobulin (A2M), pregnancy zone protein (PZP), CD109, and PZP-like A2M domain-containing 8 (CPAMD8) (52). Already in the cnidarians, the C3, A2M, and CD109 did appear as separate proteins (52). The primary sequence identity is often very low between the complement components found in invertebrate species and mammals; however, the domain structure seems to be relatively well-conserved (52). Factor B (Bf), which is involved in the alternative pathway of activation, has also been found in cnidarians and in some molluscan species but seems to have been lost in other molluscs (53).

Lamprey and hagfish seem to lack the terminal components of the complement system, forming the membrane attack complex (MAC), which is composed of the C6, C7, C8, and C9 components of the complement system of jawed vertebrates (54). Interestingly, similar to the convergent evolution of the adaptive immunity in jawless and jawed vertebrates that will be discussed later, in lamprey, another protein is used to obtain a membrane attack complex with a similar function as the C9 of jawed vertebrates (54). This protein has been identified as a natterin family member, lamprey pore-forming protein (LPFP), with a jacalin-like lectin domain and an aerolysin-like pore-forming domain (54). This protein has a high affinity for mannan and can form oligomers in the presence of mannan (54). The LPFP seems to be as efficient in the killing of target cells as the C9 complex (54).

Interestingly, a C9 homolog has been identified in colonial tunicate (*Botryllus schlosseri*), suggesting that the lytic pathway was present during early cordate evolution, which also indicates that this was lost and replaced by the LPFP in the agnathans (lamprey) (35). Interestingly, all the components downstream of C5, i.e., the C6, C7, C8, and C9, belong to the same gene family, all containing the LDLA and MACPF (MAC–perforin) domains, and some also contain additional protein domains (55).

The complement components C3, C4, and C5 are close homologs, which most likely appeared by gene duplications of a prototype C3 gene in an early jawed vertebrate (52). The same seems to be the case for factor B (Bf) and C2, where Bf is found in lamprey and molluscs but not C2, and where C2 seems to have appeared by a gene duplication of Bf in the evolution of jawed vertebrates (56).

In lamprey, C1q, the mannose-binding lectin, C3, and Bf are all present (56). In molluscs, a large number of C1q domain-containing proteins have been identified, and at least one that, like human C1q, also contains a collagen domain has been described (53). Many of these C1q domain-containing proteins have been shown to interact with bacterial PAMPs, indicating that the original role of C1q was to bind PAMPs before the emergence of immunoglobulins (53). In molluscs, proteins that function as human MBL and ficolin-like molecules have been identified, where the latter bind to various microbes and/or PAMPs (53).

The classical serine proteases of the classical and lectin pathways, C1s, C1r, MASP, and Bf/C2, have not been conclusively identified in molluscs. However, several serine proteases with similar additional domains to the mammalian counterparts have been identified, indicating that there may be some proteins with similar functions in molluscs (53).

Numerous MACPF domain-containing proteins have also been found in molluscs similar to mammalian C9. However, whether they are involved in membrane attacks, like C9, has not been investigated (53).

In some invertebrates, the number of proteins containing complement domains has increased dramatically, indicating that the complement system has taken a bigger role in immunity when an adaptive immune system is lacking or very limited in variability. In a mollusc, the Pacific oyster (*Crassostrea gigas*), the genome contains 784 complement-related genes (53). The function and role in immunity of these components are still poorly characterized, but their extremely high numbers indicate that the complement system plays a very central role in the innate immunity of these species (53).

In vertebrates, the complement system also plays an important role in the activation of B cells and may also be directly involved in T-cell responses, indicating the importance of the complement system in coordinating innate and adaptive immune responses (51).

The evolution of the complement system is apparently very complex, involving numerous gene duplication events both in early evolution, as seen with the C3-related proteins in Cnidaria, and at later stages with the appearance of the jawed vertebrates. Alternative proteins have also been involved in forming parts of the system, as seen in the jawless fishes, where other protein families are involved in the later stages of the membrane attack complex formation, as an alternative to C6, C7, C8, and C9 of jawed vertebrates.

**Figure 4:**
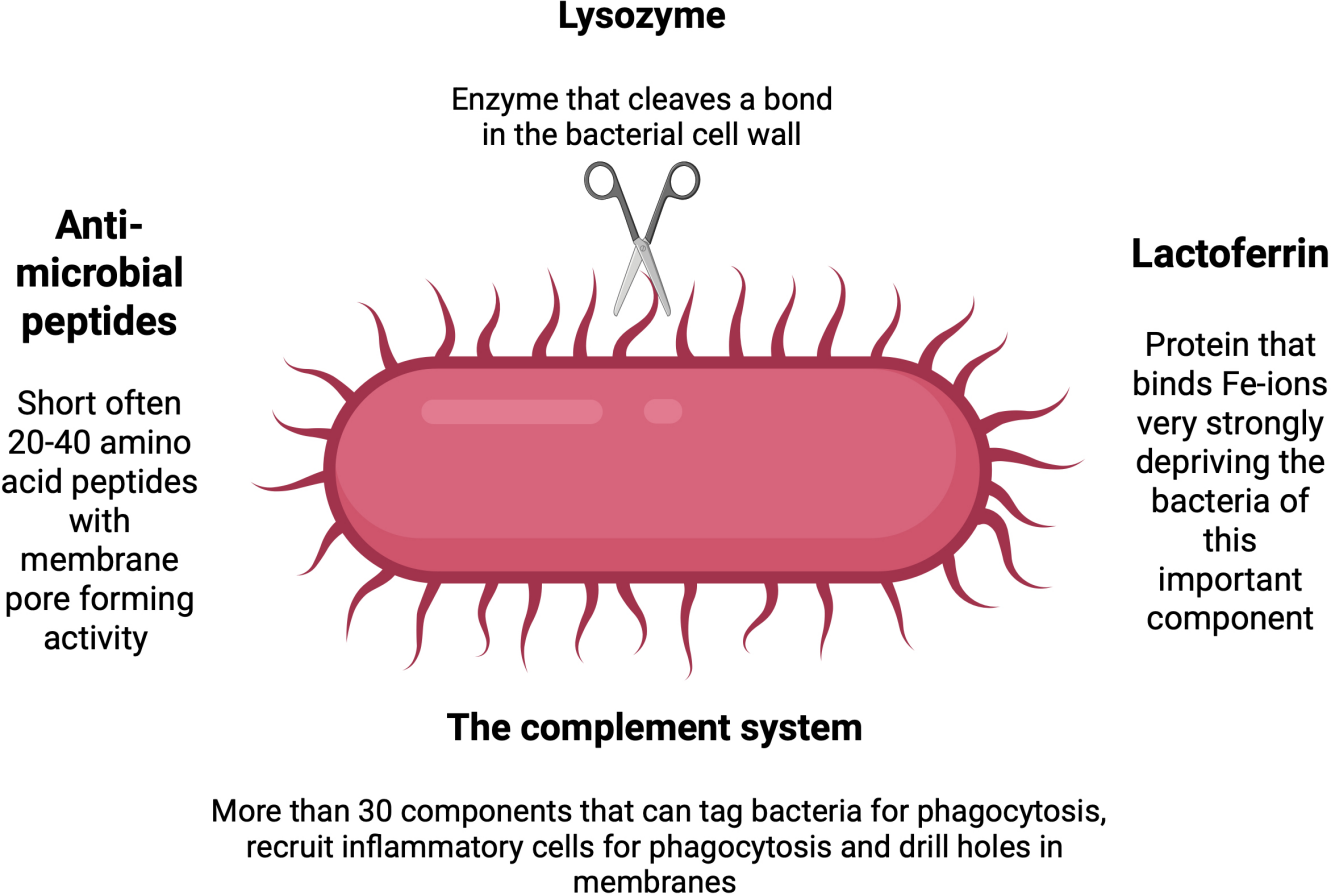
Innate immune mechanisms of multicellular organisms. Several major classes of proteins take part in innate immunity in multicellular organisms. Four of the major classes are listed in this figure, beginning with members of the large family of lysozyme-related enzymes, which cleave a specific bond between two carbohydrate moieties in the bacterial cell wall and thereby strip the bacteria of their cell wall, as a protection against bacterial infections. To this can be added various antimicrobial peptides acting on bacterial, fungal, and viral membranes and thereby, as one of their mechanisms, forming holes in the membrane. Iron-binding proteins, including lactoferrin, limit the access to this important metal ion for many, mainly redox enzymes. To these innate mechanisms can also be added the large group of proteins belonging to the complement system. The complement system has three major functions: to mark infectious agents for phagocytosis by opsonization, to attract immune cells by chemotaxis, and to form pores in the membranes of the infectious agent. The figure is made in BioRender.

A summary of the major innate mechanisms in multicellular organisms is found in **Figure 4**.

### 3.7 The coagulation system

The coagulation system can limit pathogen spread, support killing, and aid in tissue repair. However, overactivation can lead to thrombosis and also to bleeding due to the overconsumption of hemostatic factors (57). In some invertebrates, the coagulation system is part of the immune system by encapsulation and recognition of an incoming intruder and is also tightly connected to the pro-phenoloxidase system, which will be described in the next section (58, 59). In *Drosophila*, the fruit fly, the coagulation system initiates the closure of a wound by forming a clot that stops the leakage of tissue fluid and attracts hemocytes, which initiate the deposition of melanin by the pro-phenoloxidase system, which traps potential incoming pathogens at the site of injury (58).

### 3.8 The pro-phenoloxidase system

The majority of the above innate systems, both molecular and cellular, are found in most multicellular eukaryotes. However, some systems are found only in some parts of the animal kingdom. The encapsulation of incoming pathogens by the pro-phenoloxidase system belongs to this category. This system has primarily been found in crustaceans and insects (60, 61). However, indications for its presence in *Cnidaria* have come from studies of the hydra and corals, suggesting an earlier appearance of this system (62). The phenoloxidase system is based on the phenol rings of the amino acid tyrosine and of dopamine and a set of enzymes, including the phenoloxidase, to form melanin to trap incoming pathogens (60, 61, 63). This system is also involved in the healing of wounds. In *Drosophila*, three pro-phenoloxidase (PPO) genes have been identified, where at least two of them, PPO1 and PPO2, seem to be directly involved in immunity against Gram-positive bacteria and fungi, showing the importance of melanization in insect host defense (60). In contrast, the third of these genes, PPO3, seems to be involved in the defense against parasitic wasps (60). The pro-phenoloxidase system is triggered by pattern recognition receptors recognizing different compounds from bacteria and fungi, such as peptidoglycan, LPS, and fungal cell wall components, which, in turn, results in the activation of a serine protease cascade and the cleavage of the pro-phenoloxidase. The cleavage transforms this inactive pro-form into an active phenoloxidase, which starts depositing melanin on the surface of the incoming pathogen. During this activation process, the system generates oxygen radicals and NO, which actively participate in the killing of the infectious agent. The melanin also results in the accumulation of immune cells, the encapsulation of incoming bacteria or fungi, and, finally, their death. This serine protease cascade is tightly regulated by several protease inhibitors, where reduced levels of them can result in massive uncontrolled melanin formation and death. The PPO system works in close contact and in parallel with the coagulation system, as described above, which also results in the healing of wounds and in the entrapment of incoming pathogens, and where transglutaminases participate in stabilizing the blood clot (59). Interestingly, both prokaryotic and eukaryotic pathogens have developed ways to counteract the PPO system by producing protease inhibitors and other inactivators of the system, showing the major importance of this system for arthropod immunity (63). Moreover, the PPO system exhibits a variety of other activities independent of melanin production, including cytokine, opsonin, and antimicrobial activities, which further illustrates the importance of this system (61).

### 3.9 The Argonaute system in eukaryotes

Argonaute proteins are important players in bacterial immunity against phages and in anti-viral immunity via virus-specific siRNAs in plants, fungi, and invertebrates (64–68). Compared to the many questions remaining concerning the mechanisms of the Argonaute system in bacteria as described earlier, this process is relatively well-characterized in vertebrates. Two RNase III variants, first Drosha in the nucleus and then Dicer in the cytoplasm, trim double-stranded RNA into the short sequences used by the eukaryotic Argos (eAgos) (14). In contrast to other organisms, where the Argonaute system plays a very important role in immunity, in mammals and most vertebrates, it seems to play a minor role in immunity and instead seems to be primarily involved in post-transcriptional gene regulatory events (68). The short RNA sequences generated by Drosha and Dicer are used by the eAgos to target mRNA for gene regulation by blocking translation or initiating targeted mRNA degradation. The existence of three separate conserved lineages of Argonaute proteins has been identified: the siRNA class AGO, the miRNA class AGO, and the PIWI Argonaute (68). The miRNAs show high sequence conservation, whereas the siRNAs are characterized by a high rate of molecular evolution, most likely due to an arms race with viruses (68). Vertebrates seem to entirely lack the siRNA class AGO proteins, which is in line with the minor role (if any) of Argonaute proteins in viral defense in vertebrates (68). Recently, additional functions have been added to the potential function of the Argonaute system, such as development, RNA processing, and genome stability (65, 69).

As mentioned above, in contrast to vertebrates, the Argonaute proteins have an important role in immune defense in plants, fungi, and invertebrates by cleaving or silencing mRNAs by binding and thereby interfering with translation (68, 70, 71). In different plant species, there are a relatively large number of members of the Argonaut family proteins, where the majority are involved in viral or bacterial defense (70). There are 10 in *Arabidopsis*, 15 in poplar, and 17 in maize (70). In contrast to plants, only one member is found in fission yeast (*Schizosaccharomyces pombe*), five in fly (*D. melanogaster*), and 27 in worm (*C. elegans*) (70). The Argonaut 2 protein in *Drosophila* has been found to be essential for its anti-viral immunity (67). Interestingly, fungal short RNAs (RNAi) can be transferred to plant cells to silence immune genes, enhancing fungal plant infection (71). They can thereby act on both immunity and virulence factors.

## 4 Innate cellular immunity in multicellular eukaryotes

In addition to soluble components, there are several types of cells that have become an important part of the innate immune system, and some of them are found in almost all multicellular organisms.

### 4.1 Macrophages and monocytes

**Figure 5:**
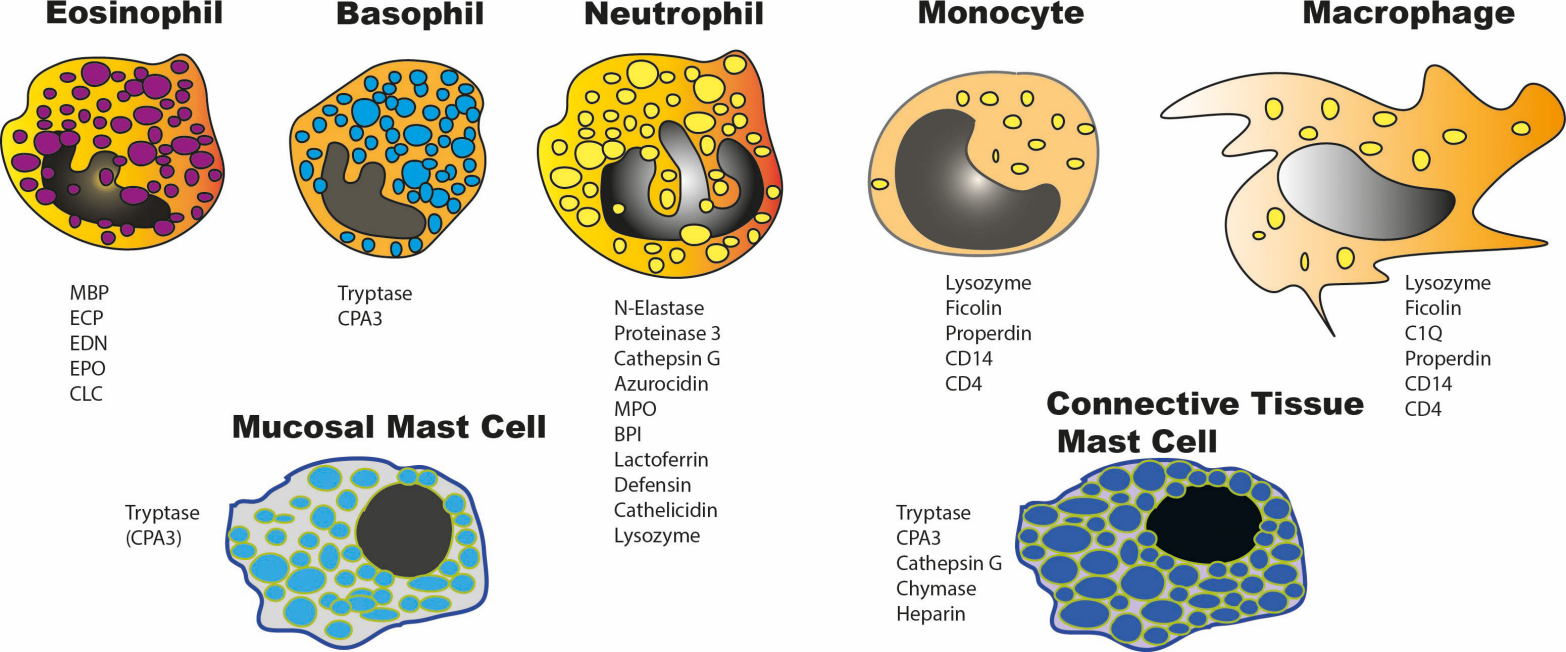
Human innate immune cells. The figure shows schematic pictures of seven different human innate immune cells, including the three polymorphonuclear granulocytes, the eosinophil, the basophil, and the neutrophil, followed by the monocyte, the tissue macrophage, and the two types of mast cells, the mucosal mast cell and the connective tissue mast cell. The three granulocytes and the monocyte are primarily located in the blood, whereas macrophages and mast cells are tissue cells. Major proteins stored in granules of these cells or expressed on their cell surface as receptors are listed below or on the left side of these cells in the figure. Major basic protein (MBP), eosinophil cationic protein (ECP), eosinophil-derived neurotoxin (EDN), eosinophil peroxidase (EPO), Charcot–Leyden crystal protein (CLC), carboxypeptidase A3 (CPA3), myeloperoxidase (MPO), and bactericidal permeability-increasing protein (BPI). The figure is made in Adobe Illustrator.

One such cell is the macrophage. Macrophages and macrophage-like cells act as highly mobile scavengers that can attack and ingest incoming microbes and kill and degrade them inside the cell in phagosomes (**Figure 5**) (72, 73). They are also involved in removing apoptotic cells and immune complexes of antibodies and foreign material, including dead bacteria and viruses (73). Macrophage cells have been found at the root of multicellular organisms, as represented by sponges. However, they also seem to be absent in some multicellular eukaryotes, such as the nematode *C. elegans*, where the removal of apoptotic cells is performed by neighboring cells (74). In mammals, the majority of tissue macrophages primarily originate from two early waves of cells from yolk sac-derived cells and are subsequently maintained independently of adult hematopoiesis (75–77). These cells maintain their proliferative potential in the periphery and can increase in numbers by cell division if cells have been damaged and have died during an inflammatory response. Monocytes originating from the adult bone marrow seem primarily to act as inflammatory cells and to be able to enter inflamed tissue, where they can differentiate into macrophage-like cells (**Figure 5**). When the inflammation/infection has been resolved, most of these monocyte-derived macrophages seem to disappear, most likely by apoptosis. It is only in the intestinal region and the lungs where monocytes seem to play a major role in generating tissue macrophages (78). In these regions, there is a high turnover of cells, which may be the reason why we have macrophages at these anatomic locations that are monocyte-derived, as monocytes can be recruited from the circulation.

Macrophage-like cells may actually be the ancestor of all cells in multicellular organisms, as discussed in a recent article on the origin and functional versatility of macrophages (73). The first multicellular organisms may have originated from free-living amoebas that, like *Dictyostelium discoideum*, form a type of multicellular organism in response to starvation, where cells in the fruiting body differentiate into four different cell types (73). The free-living *Acanthamoeba* hunts microbes for nutritional needs and shows similar size and behavior to mammalian macrophages. They also react by chemotaxis towards the microbes initiated by the binding of bacterial products to pattern recognition receptors similar to inflammatory M1 macrophages (73). Among the most primitive multicellular organisms are sponges, which lack fully differentiated tissues and only contain a few major cell types (73). The filtering body lacks nervous, digestive, and circulatory systems and is formed by a gel-like matrix called mesohyl, which is sandwiched between two layers of cells, the outer pinacoderm and the inner choanoderm (73). The choanocytes have flagella that generate water flow and absorb nutrients by phagocytosis (51). The nutrients are then distributed to other cells by mobile macrophage-like archaeocytes. These archaeocytes seem to be the only cells of sponges that have preserved multipotency and have the capacity to fully generate the entire body of sponges when separated as individual cells (73).

Mammalian macrophages have, in addition to their major role in phagocytosis, a potential role in the regulation of both the coagulation and complement systems by expressing several components of both systems. Macrophages may therefore be an important local producer of these components in addition to the liver, which produces the majority of the coagulation and complement components found in the circulation (79).

### 4.2 Polymorphonuclear granulocytes and mast cells

In addition to the macrophages, there are also other innate immune cells. In humans, there are three different polymorphonuclear granulocytes—neutrophils, basophils, and eosinophils—and the tissue-resident mast cells (**Figure 5**). The cytoplasm of these cells is filled with electron-dense granules. The contents of these granules, together with their surface receptors, give these cells their primary function in immunity (**Figure 5**).

Neutrophils are very important for defense against bacterial infections, and their granules contain several antibacterial molecules, including the antimicrobial peptides defensin and cathelicidin, the antibacterial enzyme lysozyme C, and the iron-binding protein lactoferrin. They also store the hypohalide-generating enzyme myeloperoxidase (MPO), the lipopolysaccharide-binding bactericidal permeability-increasing protein (BPI), and several very abundant serine proteases, including N-elastase, proteinase 3, cathepsin G, and azurocidin (80, 81). Some of these proteases have direct antibacterial activity.

Eosinophils have several proteins with functions in parasite defense, such as major basic protein (MBP), eosinophil cationic protein (ECP), eosinophil-derived neurotoxin (EDN), and also the hypohalide-generating enzyme eosinophil peroxidase (EPO) (82). EPO is closely related to MPO and TPO, a third thyroid-specific peroxidase. These three enzymes most likely appeared by gene duplication. Both EPO and MPO show a preference for bromide and chloride when generating hypohalide ions (82).

Basophils seem primarily to act as inflammatory cells, producing significant levels of cytokines, including the Th2-inducing cytokines IL-4, IL-13, and IL-25 (83–86). The granule content of human basophils is less well-characterized but contains proteoglycans, primarily chondroitin sulfate E, and a few proteases, including tryptase and carboxypeptidase A3 (87, 88). Both these enzymes are also found in larger amounts in granules of mast cells (33). Mouse basophils differ significantly in granule content from human basophils by having different proteases. Mouse basophils express two basophil-specific proteases, mMCP-8 and mMCP-11, and the carboxypeptidase A3, with the latter also expressed by mouse mast cells (83, 89–91).

Mast cells are hematopoietic tissue-resident cells that also belong to the innate immune system. Two types of mast cells have been identified in mammals: the connective tissue mast cells (CTMCs) and the mucosal mast cells (MMCs) (**Figure 5**). Both are located in areas of close contact with the external world, such as the skin, the tongue, the lung, and the intestinal mucosa, where they can act as guardians against external threats (92). Both types of mast cells have, similar to granulocytes, their cytoplasm filled with electron-dense granules. The two types of human mast cells contain partly different granule material. They both contain heparin and chondroitin sulfate proteoglycans, as well as proteases, but the type of granule proteases differs between the two subpopulations. MMCs only contain tryptase and are therefore commonly termed MC_T_ in humans, whereas CTMCs, also termed MC_TC_, contain tryptase, chymase, cathepsin G, and carboxypeptidase A3 (CPA3) (33). Both granulocytes and mast cells have primarily been characterized in placental mammals. However, similar cells are found in many vertebrates (93, 94). A mast cell-like cell has even been found as early as in a tunicate, an early chordate, the sea squirt (95). Although there are large similarities between mast cells from different species, we also see major differences. Mast cells from rats and mice differ significantly in their granule content from human mast cells. In mice, it is only CTMCs that contain heparin, whereas MMCs contain the less negatively charged chondroitin sulfate (96, 97). The CTMCs of mice and rats also almost totally lack cathepsin G, the neutrophil protease that is the most highly expressed protease gene in human CTMCs (33, 92). Mouse MMCs, which are found in the duodenum, also lack the tryptase and express only two chymases, the mMCP-1 and mMCP-2 (89). Therefore, human MMCs only contain one tryptase, and mouse MMCs only contain two chymases, of which only one seems to be enzymatically active, the mMCP-1 (98, 99). A mast cell-like cell has also been identified in zebrafish, which contains a carboxypeptidase similar to that of mammalian CTMCs. However, the zebrafish carboxypeptidase is not a direct homolog of the mammalian CPA3, indicating convergent evolution (94, 100). Interestingly, the tunicate mast cell-like cells contain a trypsin-like enzyme, histamine, and heparin, thereby showing similarity to mammalian mast cells, indicating that mast cell-like cells appeared in early chordates (95).

When it comes to the evolution of the different granulocytes, a clear picture is sometimes difficult to obtain due to a lack of phenotypic characterization of the cells. Although visual observation and staining of cells show characteristics similar to the mammalian counterparts, a detailed analysis of their proteome or transcriptome is often lacking. In chicken, we find relatively high numbers of a cell type that resembles mammalian neutrophils. This cell type, named heterophils, has only been partly characterized concerning its granule content, which is why a more detailed analysis of its similarities and differences to the human neutrophils is still not possible (101). Both basophil and eosinophil-like cells have also been identified in chickens (102, 103). However, almost nothing is known about their role in chicken immunity.

### 4.3 Granule proteases

**Figure 6:**
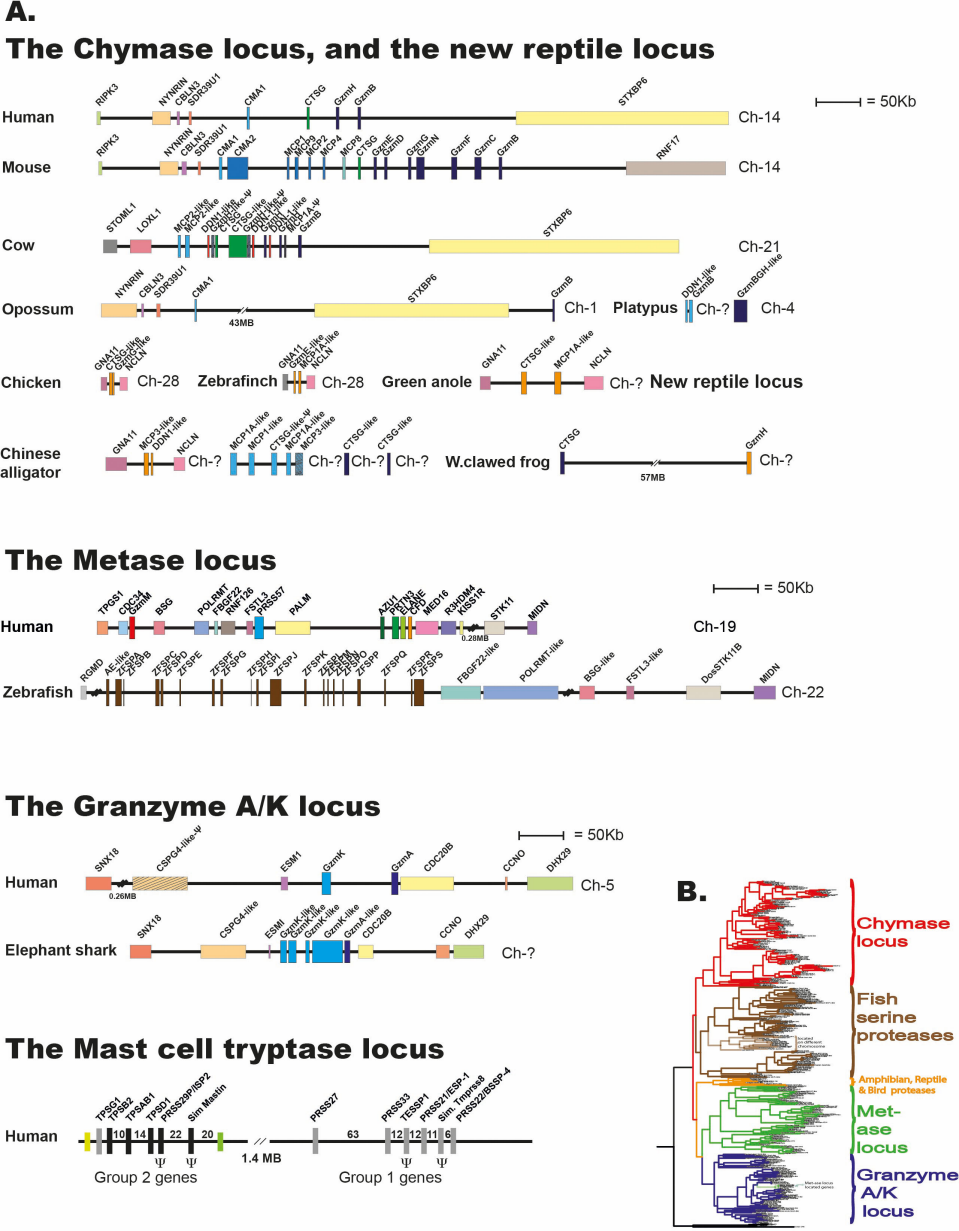
Hematopoietic serine protease loci. **(A)** shows the five major loci encoding hematopoietic serine proteases, the chymase locus, the new reptile locus, the Met-ase locus, the granzyme A/K locus, and the mast cell tryptase locus. **(B)** shows a phylogenetic tree of several proteases from each of these four loci and how they cluster in separate branches. In contrast to the tetrapod proteases, which fall into four separate branches, most of the fish proteases, even when found in the same loci, form a separate branch, indicating that they have evolved separately from the tetrapod proteases, even if they are found in the same locus, as based on similar bordering genes. The figure is made in Adobe Illustrator.

As mentioned above, massive amounts of the granule proteases are found in several of the above-listed cell types, including mast cells, neutrophils, and basophils, but also in cells of the lymphoid lineage, the cytotoxic T cells, and NK cells. The evolution of these proteases is complex. In tetrapods, these proteases are encoded in five different loci, and only one of them is found in cartilaginous fishes, the granzyme A/K locus (104) (**Figure 6A**). None of them is found in jawless fishes, indicating that they first appeared in jawed vertebrates (104). A similar genomic organization is seen in different tetrapods. However, the expansion of these proteases seems to have taken another route in fish, as most of the hematopoietic serine proteases in fish form a separate branch in a phylogenetic tree (**Figure 6B**) (104). Many different functions of these proteases have been found, but they are not always easy to clearly define, as they can have a large number of potential substrates, where the biological significance of the cleavage can sometimes be difficult to interpret. Neutrophil proteases may primarily be involved in the migration of neutrophils through thick connective tissue to reach the area of infection and in the defense against bacteria. They are very abundant and very active, and uncontrolled release can result in severe tissue damage, including lung emphysema (105). None of the neutrophil proteases is expressed in T cells or NK cells (33). Instead, these cells have granzymes (33, 80, 88). In humans, there are five different granzymes, A, B, H, K, and M, which are all expressed almost exclusively in T cells and NK cells (33). The most well-characterized is granzyme B, which is important for the induction of apoptosis by cytotoxic T cells (58). A much less clear picture has been obtained for the other granzymes. However, results indicate that several of them are also important for the induction of apoptosis in cells infected with intracellular parasites such as viruses (80). Mast cell proteases have, in contrast, been found to be involved in a large number of inflammatory processes, including tissue remodeling by cleavage of fibronectin and other connective tissue components (106). They are involved in increasing intestinal permeability by cleavage of occludin, cadherins, and other adhesion molecules of intestinal epithelial cells (107). Both chymases and tryptases are important for protection against snake, scorpion, and Gila monster venoms and as a negative regulator of Th2 immunity by cleaving a highly selective set of Th2-inducing cytokines and chemokines (108–111). The chymases of CTMCs have also been found to cleave the anticoagulants of some blood-feeding parasites and, by efficient cleavage of angiotensin I, to generate angiotensin II (112–114). The generation of angiotensin II indicates a role of mast cell chymases in blood pressure regulation (113, 114). Studies using knock-out and transgenic animal models, or studies using cleavage analysis of pure recombinant enzymes and potential targets from tissues or body fluids, will most likely result in additional targets of interest. Therefore, the list of potential functions of these very abundant proteases is still increasing.

When it comes to hematopoietic serine proteases in species other than placental mammals, recent studies have shown that the apoptosis-inducing enzyme, granzyme B, is one of the first to appear in the chymase locus and that this enzyme has a very similar cleavage specificity in marsupials, monotremes, and even the Chinese alligator (115–118). A protease with expected granzyme B characteristics is also found in the chymase locus of the clawed frog (118). The mast cell chymase is also found in both marsupials and monotremes but seems not to be present in frogs, indicating that it appears somewhat later during tetrapod evolution (116, 119). Two immune proteases with yet unknown functions have also recently been identified and characterized concerning their cleavage specificity and tissue distribution in an early branch of fish evolution, the spotted gar (120). An immune protease that may have a similar function to mammalian granzyme B has also been characterized in the channel catfish (121). The collected information from fish and tetrapods indicates that fish have experienced a parallel evolution of the hematopoietic serine proteases compared to the tetrapods, except for the proteases of the granzyme A/K locus, where we see direct homologs from cartilaginous fishes to humans (122–124).

### 4.4 Immune cells in invertebrates

A relatively detailed characterization of immune cells has also been performed in a few invertebrate species. In the fruit fly, *D. melanogaster*, three types of immune cells involved in cellular immunity have been described: plasmatocytes, crystal cells, and lamellocytes. Plasmatocytes are professional phagocytes involved in the phagocytosis of invading microbes but also producers of both AMPs and coagulation factors (125). In contrast, the crystal cells are non-phagocytic and instead are involved in the melanization reaction. They contain large amounts of the phenol oxidase enzyme in crystalline inclusions, which can be rapidly released upon the activation of the cell, as regulated through the JNK pathway (125). The lamellocytes are specialized cells only formed at the infection by parasitic wasps or upon wounds in the larval stage of the fly. They act together with the plasmatocytes in the protection against parasitic wasps. The plasmatocytes first adhere to the surface of the parasite eggs. This is followed by the attachment of lamellocytes that form a multicellular layer on top of the parasite eggs. The capsule formed around the parasite egg is then melanized; however, the role of crystal cells in this process is yet unclear (125). In addition to the cellular immune response described above, the *Drosophila* fat body contributes to the humoral response by being a major producer of AMPs (125). Work has been performed in many other species. However, due to the size of this communication, we limit the description to this single invertebrate species.

## 5 Adaptive immunity in jawed vertebrates

### 5.1 Adaptive immunity

In vertebrates, adaptive immunity has evolved as a complement to innate immune mechanisms, enabling a more effective response to infections. One of the key features that distinguish the adaptive immune system from the innate system is immunological memory. Whereas the innate immune system responds in a similar way to repeated infections, the adaptive immune system responds faster, with greater specificity and with increased strength upon subsequent encounters with the same pathogen. This enhanced secondary response is the basis for vaccination, which prepares the immune system for future infections.

In jawed vertebrates, the adaptive immune system is built on several essential components: Igs or antibodies, TCRs, antigen presentation by major histocompatibility complex (MHC) molecules, and a large number of additional cell-surface molecules. Igs and TCRs are expressed by specialized immune cells known as lymphocytes. During infection, lymphocytes expressing an Ig or a TCR with specificity for the invading antigen are selectively activated. Antigen recognition delivers a proliferative signal, causing these cells to expand in number and thereby contribute to efficient elimination of the pathogen—either through antibody-mediated mechanisms or through cellular immune responses.

This type of adaptive immune system is unique to jawed vertebrates. In contrast, jawless fishes possess an adaptive immune system with similar overall function and organization but based on entirely different molecular components. Instead of Igs and TCRs, they rely on variable lymphocyte receptors (VLRs), which serve analogous roles but derive from a completely different protein family characterized by leucine-rich repeat (LRR) structures.

### 5.2 Immunoglobulins

**Figure 7:**
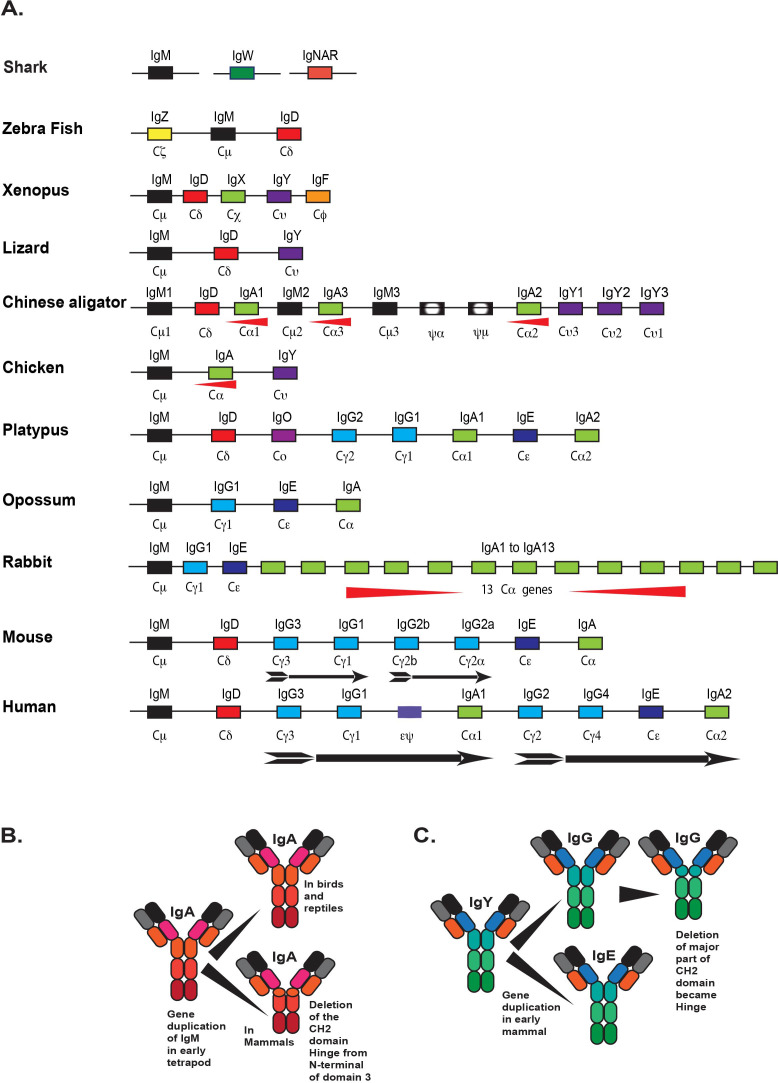
The immunoglobulin heavy-chain locus of several jawed vertebrates and the evolution of the hinge region in IgA and IgG of mammals. In **(A)**, only the constant domains are displayed. The variable regions, the D and J segments, are positioned upstream of the IgM μ gene. Gene names and the Igs they produce are marked below and above the genes, respectively. Red arrows mark the different transcriptional orientation of the IgA genes in chicken and Chinese alligator, and some of the duplicated IgA copies in the rabbit, which are in an opposite transcriptional orientation compared to the first IgA genes in the rabbit. Black arrows mark the duplicated regions in the mouse and human Ig heavy-chain loci. **(B, C)** shows the evolution of the hinge region of IgA and IgG and the gene duplication of IgY that resulted in mammalian IgG and IgE. The figure is made in Adobe Illustrator.

Adaptive immunity in jawed vertebrates is primarily mediated by soluble antibodies (Igs) and by cellular immune responses carried out by cytotoxic T lymphocytes (CTLs). Immunoglobulins are produced by B lymphocytes, while the activation and clonal expansion of both B lymphocytes and CTLs are regulated by CD4^+^ T helper (Th) lymphocytes. Th cells are, in turn, activated by antigen-presenting cells and regulated at least partly by innate lymphoid cells (ILCs) and by cytokines and chemokines coming from tissue-resident and antigen-presenting cells. Igs and TCRs are present in all jawed vertebrates, including cartilaginous fishes, ray-finned fishes, amphibians, reptiles, birds, and mammals (**Figure 3**). However, the number of Ig classes, the number of genes within a class, and the different isotypes vary greatly between different species (**Figure 7**). In cartilaginous fish, such as sharks and rays, there are, in general, three classes: the IgM, IgD/W, and IgNAR (**Figure 7**) (126). There are also generally three classes in ray-finned and bony fishes, five classes in amphibians, and five classes in humans, but in humans, these have expanded into nine isotypes (**Figure 7**). Relatively large differences in the number of isotypes within an evolutionary lineage have been observed. For example, the American alligator has three Ig classes but several copies of each of them and thereby 10 different isotypes, whereas some birds and a lizard, which also have three classes, IgM, IgA, and IgY, only have one gene for each class and thereby have a total of only three isotypes (**Figure 7**). The number of Ig genes can vary greatly between species, even within a group of animals. Independent gene duplications have most likely contributed to the large difference in the number of isotypes between even closely related species. In mice and humans, a first gene duplication resulted in two IgG isotypes, followed by a second, larger duplication that in the mouse genome resulted in four IgG isotypes. In humans, this second duplication also included the genes for IgE and IgA, resulting in four IgG genes, two IgA genes, and two IgE genes. However, one of the genes for IgE has experienced a deletion of exon 1 and has therefore become an inactive pseudogene. In the rabbit, several successive gene duplications, only involving the gene for IgA, have resulted in 13 IgA genes, where several are in reversed orientation (**Figure 7A**).

In addition to the varying number of isotypes between species, a gradual increase in both the number of classes and isotypes has occurred during vertebrate evolution. One of the classes, the mucosal Ig, the IgA, most likely appeared with tetrapods by a gene duplication of IgM. IgA has been named IgX in frogs, and IgA and IgX have been found in reptiles, birds, and amphibians. All of them have four constant domains similar to those of IgM. However, in mammals, the C2 domain has been lost and has been replaced by a hinge region (**Figure 7B**). This most likely occurred during early mammalian evolution, as this deletion is found in IgAs of all three mammalian lineages, the monotremes, the marsupials, and the placental mammals (127–129). The presence of a hinge increases structural flexibility for the antigen-binding parts of the antibody to bind multimeric repeated antigens on the surface of a virus or bacteria at different distances due to the possibility of adjusting the width between the two arms of the antibody.

Approximately at the same time as the appearance of IgA, another antibody class appeared, the IgY. IgY has also been suggested to originate from a gene duplication of IgM. IgY is found in amphibians, reptiles, and birds but not in lungfish, which is considered the direct ancestor of tetrapods (130). IgY seems, therefore, to have appeared with the tetrapods. During early mammalian evolution, IgY duplicated, and two of the copies became the ancestors of mammalian IgG and IgE (**Figure 7C**). During early mammalian evolution, one of the newly generated copies of IgY lost the CH2 domain and became present-day IgG, whereas the second copy became IgE. In contrast to IgG, IgE has in mammals four constant domains, similar to bird, reptile, and amphibian IgY (**Figure 7C**) (127, 131). In the platypus, a monotreme, a third copy of IgY is found. This extra copy is most likely also the result of the early gene duplication event that resulted in the ancestors of both IgG and IgE. This extra copy in the platypus has been named IgO (132). This gene appears to be expressed only at very low levels in the spleen and may be reminiscent of the gene duplication without a major function (132). In all three mammalian lineages, the second domain of IgG was not totally lost but transformed into a flexible short hinge region. As mentioned above, the hinge regions of IgG and IgA made the antibodies more flexible to bind multimeric repeated antigens.

The notion that IgY and IgA originate from a gene duplication of IgM has recently been challenged by the analysis of homology between different domains of these Ig classes and of IgM and IgW in cartilaginous fishes. The new analysis indicates that both IgM and IgW have been contributing to the evolution of both IgY and IgA (130). The constant domain 1 shows major similarities to both IgM and IgW, whereas domain 2 is the most homologous to IgW, and domains 3 and 4 to IgM (130). IgM is the Ig class that shows the highest degree of conservation during evolution, whereas IgW is much more rapidly evolving, involving several internal duplications within the constant domain (130). IgM retains what appears to be the original structure with four constant domains in all analyzed species. In contrast, IgW exists as either a seven-domain or three-domain form in cartilaginous fish, and in lungfish, it can have from two to at least 11 domains (126, 130).

### 5.3 Fc receptors

**Figure 8:**
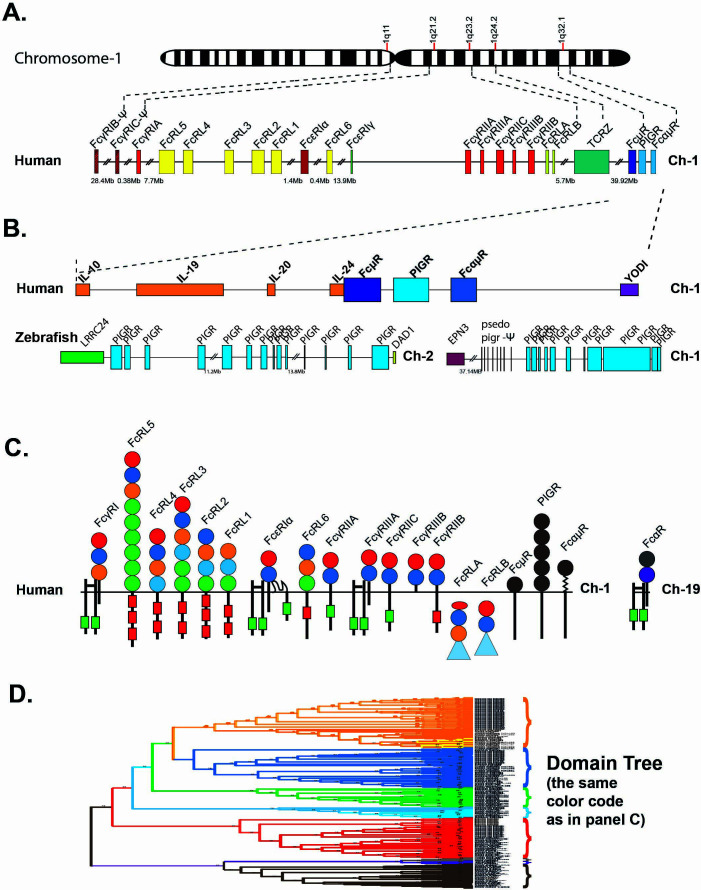
Human and zebrafish Fc receptor genes. **(A)** shows an in-scale map of the human Fc receptor locus on chromosome 1. **(B)** shows an enlarged section of the human Fc receptor locus on chromosome 1, including the IgA/M, the human IgM, and the poly Ig receptors (PIGRs), together with two regions in the zebrafish genome encoding 25 different PIGR genes. In **(C)**, the Ig-like domains are depicted as filled circles with color-coding according to the similarities in sequence based on phylogenetic analyses. The domain types D1, D2, D3, D4, and D5 show a relatively conserved pattern in most tetrapods and have therefore been color-coded in red, dark blue, yellow, light blue, and green. **(D)** shows a phylogenetic analysis of all the individual domains, using the same color code as in **(C)**. The figure is made in Adobe Illustrator.

**Figure 9:**
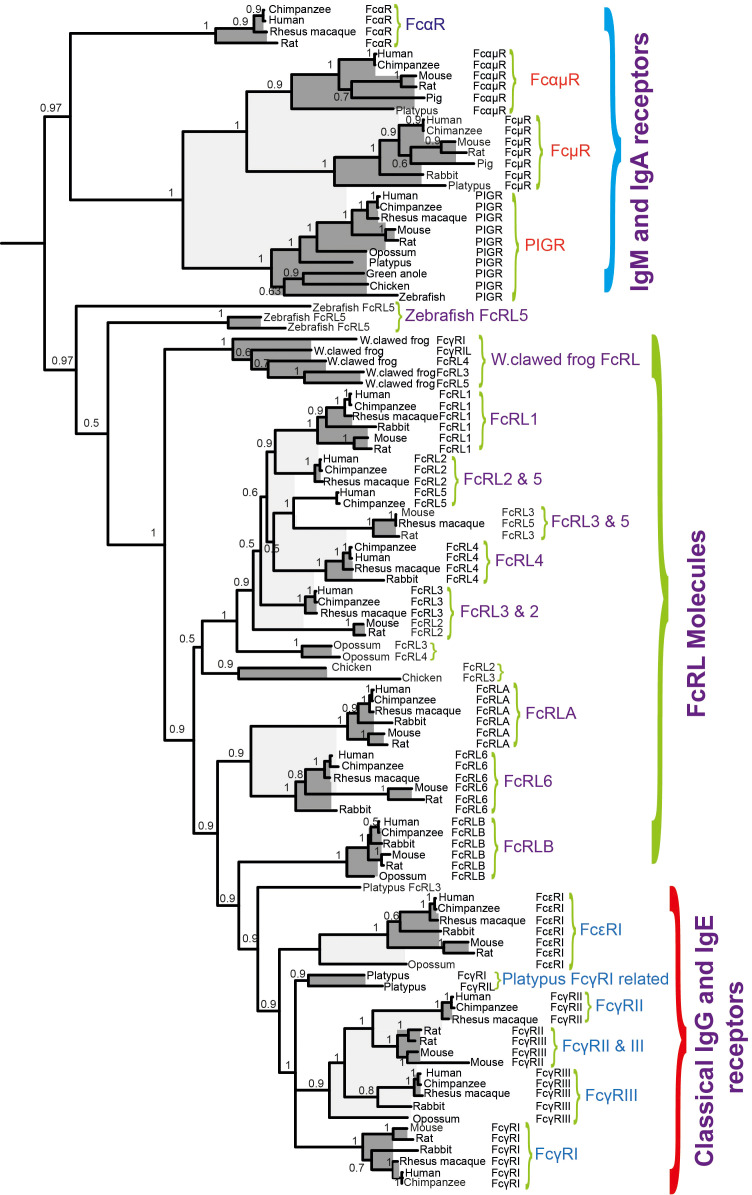
Phylogenetic tree of vertebrate Fc receptors. As can be seen from the figure, the classical mammalian IgG and IgE receptors form a separate branch on this tree. The closest relatives to this branch appear to be FC receptor-like B, A, and 6 (FCRLB, FCRLA, and FCRL6, respectively), indicating that the classical mammalian IgG and IgE receptors originate from these Fc receptor-like genes. The figure is made in Adobe Illustrator.

The increase in Ig classes and isotypes made it possible to diversify their role in immunity. One important function of antibodies is their binding to specific receptors on the surface of various immune cells. These receptors, named Fc receptors, bind to the constant domains of the antibodies, which enable these cells to act specifically to an antigen using the antibodies as an affinity ligand. These receptors can act during phagocytosis and in the activation of cells by receptor crosslinking. In parallel with the duplication event of IgY and the formation of IgG and IgE, a subclass of the Fc receptors, called the Fc receptor-like molecules, seems to have expanded in numbers by gene duplications and later developed into the classical Fc receptors for IgG and IgE (133). The specific receptors for IgG and IgE seem thereby to have appeared in parallel with the appearance of these isotypes during evolution. The genes for all of these receptors are located on chromosome 1 in the human genome (**Figure 8**) (133). Receptors for both IgM and IgA, and the dual receptor for IgA and IgM seem to have appeared during early mammalian evolution or in some cases somewhat later (134). The type and number of receptors interacting with the Igs vary greatly between different vertebrate species. For example, all the classical Fc receptors [including the poly Ig receptor (PIGR); IgG, IgE, IgA, and IgM receptors; and also the relatively recently identified Fc receptor-like molecules] are lacking in cartilaginous fishes (133). The question is, therefore, how the cartilaginous fishes manage classical phagocytic activities, which in mammals involves the classical Fc receptors, and how they transport immunoglobulins into secretions, which is performed by PIGR. Bony fishes also lack the classical Fc receptors, including the IgG, IgE, IgA, and IgM receptors, but have PIGR and the Fc receptor-like molecules (133). In some bony fishes, including the zebrafish, there has instead been a massive increase in the number of PIGR genes and also a diversification of these receptors. The single PIGR member in the human genome encodes five extracellular domains, whereas zebrafish have at least 25 such genes, where the domain number varies between two and six (**Figure 8**) (133, 134). The classical receptors for IgG and IgE seem to have appeared from a duplicated copy of FCRLB, as FCRLB is the closest homolog of these IgG and IgE receptors (**Figure 9**).

### 5.4 Variable regions

**Figure 10:**
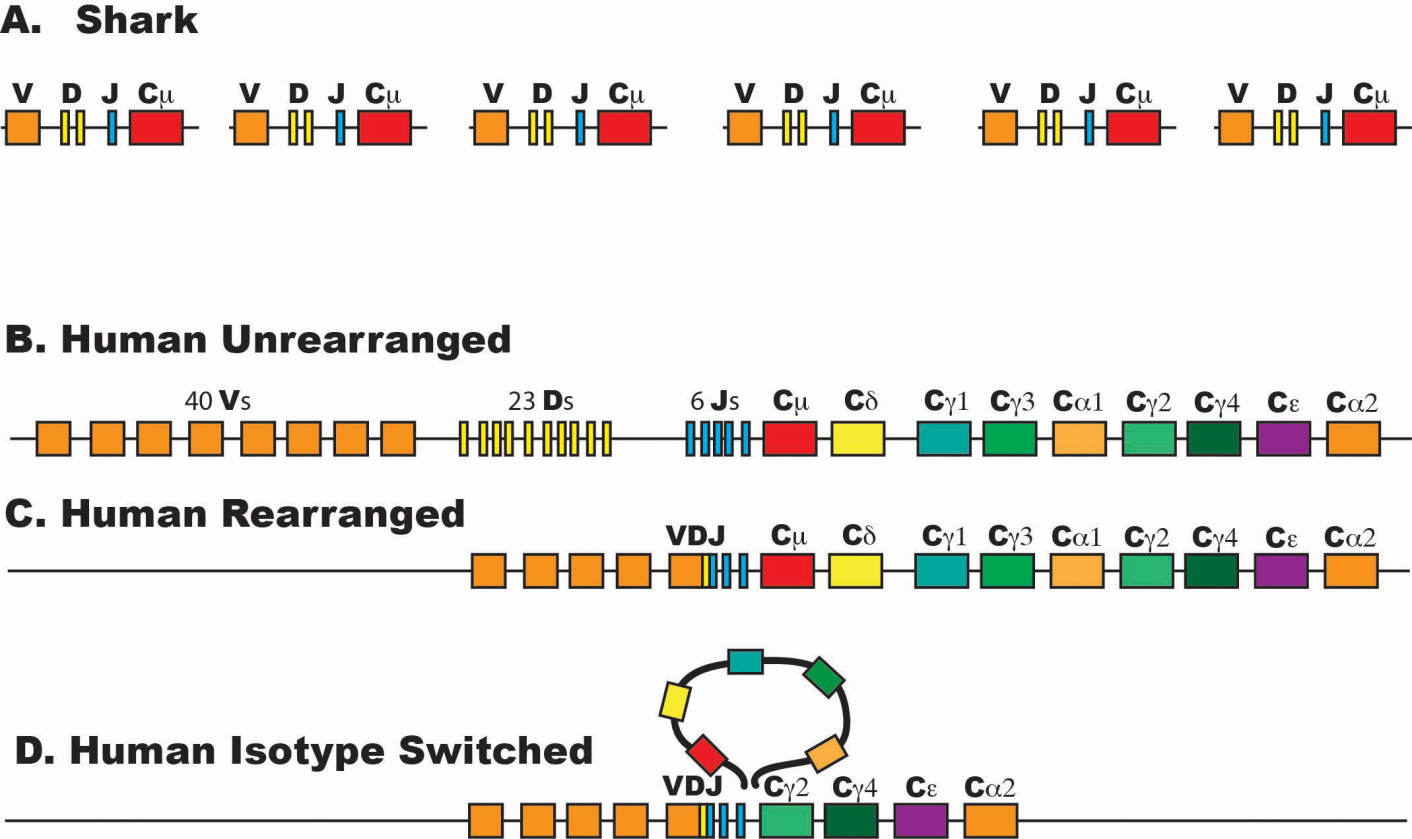
Difference between shark and human Ig loci. **(A)** shows a part of a shark IgM region with a large number of separate VDJC regions, which can rearrange independently. **(B)** shows the unrearranged human heavy-chain locus with multiple V regions, multiple D regions, multiple J regions, and multiple C regions in the same locus. **(C)** shows a rearranged locus forming a functional VDJ region sitting in front of the IgM constant region. **(D)** shows the same locus after an isotype switch to IgG2. The figure is made in Adobe Illustrator.

The variable regions of the Ig heavy chain and one of the T-cell receptor chains are formed by the ligation of three different gene segments: the variable (V), the diversity (D), and the joining (J) segments. When we look at the variable regions, there are quite large differences in the numbers of the different gene segments forming the functional variable region (**Figure 10**). Some species have many D segments and fewer J segments, and others have relatively few D segments and more J segments. A lower number of D and J segments can also be compensated for by a higher number of V segments. In the light chains and in one of the T-cell receptor chains, there are instead only two types of segments that are combined to form a functional variable region, the V and J segments.

### 5.5 Immunoglobulin locus organization

The Ig locus or loci are organized in a very different way in cartilaginous fishes and in mammals. Both IgM and IgD/IgW in cartilaginous fish are separated into a large number (50–100) of mini loci containing only one V segment, multiple D segments, a single J segment, and a single constant region (**Figure 10**). The question is how and when the multi-locus arrangement of the Ig genes, as seen in cartilaginous fishes, was transformed into a single locus, as in amphibians, reptiles, and mammals, which instead have multiple V, D, and J segments in a single locus also containing all the constant regions (**Figure 10**). It seems as if this process was gradual, as some fish species exhibit a mixed arrangement with several gene loci for a certain class of Igs. This indicates that they are in a process of initiating an arrangement with several V regions located in a cluster in front of each of several constant domains that, in several cases, such as the lung fishes, are located on several different chromosomes (130). The advantage of a single locus is that the B cells can restrict the expression of a single heavy and a single light chain, which allows for a more efficient selection and amplification process of the desired specificity by clonal selection (130). This may have been a key event in the development of larger animals with constant and high body temperatures, which are most likely at higher risk for rapid spread of infectious agents due to their higher body temperature. A coordinated switch from the original IgM to another isotype more suited for combating the infection in a certain region of the body, while maintaining the same V-gene specificity, is also a major advantage (**Figure 10**). This isotype switch seems to have appeared with tetrapods (135).

### 5.6 Variability-generating mechanisms

The assembly of a functional Ig or TCR is a complex process where the different gene fragments need to be recognized, brought together, and ligated to form a functional transcription unit. This process is dependent on two recombinases, the recombination-activating genes RAG1 and RAG2, that bring the gene segments together and facilitate the deletion of the intervening DNA sequences (136). One intriguing question in the evolution of adaptive immunity in jawed vertebrates has been the origin of the RAG1 and RAG2 genes and the separation of the V, D, and J segments from what is thought to be an originally continuous sequence. Homologs to vertebrate RAG1 and RAG2 have been found in the purple sea urchin, the Chinese lancelet, an insect, a mollusc, and even in a cnidarian, indicating a very early appearance during the evolution of multicellular organisms (137–141). The function of these RAG genes in early chordates and in invertebrates is still not known, but it seems to have been the prerequisite for the appearance of the RAG-mediated variability-generating mechanism of jawed vertebrates. The evolution of the active RAG1 and RAG2 in jawed vertebrates involves at least two different transposons. It is believed that the major part of RAG1 evolved from a transposase of the Transib superfamily and that the N-terminal part came from another transposable element, the N-RAG-TP found in the sea slug *Aplysia californica* (140, 142, 143). The RAG1/2 pair may even have been present in most metazoan lineages but lost in some lineages, such as the jawless vertebrates and some urochordate lineages (141, 144). It is possible that these genes have been introduced into the genomes by horizontal transfer several times during eukaryote evolution, but have only become a part of the variability-generating mechanism of the adaptive immune system in jawed vertebrates (143).

In addition to the variability-generating mechanism involving the RAG1 and RAG2 and the random combination of V, D, and J segments, Igs also increase their specificity for a particular antigen by single-nucleotide substitutions, primarily in the coding region of the rearranged VDJ region. This modification of a preselected V region is a mechanism for affinity maturation. Mutated V regions with higher affinity for the antigen will have an advantage when competing for antigen binding, and if successful, it will favor the survival of this particular clone by strong selection in the spleen and lymph nodes. The enzyme responsible for these single-nucleotide substitutions, which has been named somatic hypermutations, is the activation-induced cytidine deaminase (AID) (145, 146). This enzyme is also responsible for an additional process during B-cell maturation, the isotype switch. Following the assembly of the functional V region, all mammalian B cells start expressing IgM. However, IgM may not be the most suitable Ig class later during an immune response, partly due to its large size as a pentamer. The B cell can then change the Ig isotype of the selected V region by an isotype switch. The gene segment for the new isotype is then moved close to the rearranged V region so that the transcript originating from the V-region promoter includes the constant domains of this new isotype. The intervening gene region for the isotypes between the V region and the selected isotype is then deleted from the genome in a switch circle (**Figure 10**). AID belongs to a larger family of AID/APOBEC enzymes comprising the AID, APOBEC1, APOBEC2, and APOBEC3 subgroups, which deaminate cytidine in RNA and/or DNA and exhibit diverse physiological functions (145). By the isotype switch, the B cells can change the isotype to the most suitable isotype for the targeting of the infectious agent. If the agent is appearing in the intestinal region, IgA is a suitable isotype due to its capacity to move across mucosal epithelium by selective transport by the PIGR. If the antigen instead appears in the circulation, one of the IgG isotypes is then most likely a more suitable isotype. This process of selecting a particular isotype is regulated by the cytokines present in the area of antigen contact. As an example, in the intestinal region, TGF-beta is a strong inducer of the switch to IgA (147). IL-4 and IL-13 are strong inducers of a switch to IgE (148).

**Figure 11:**
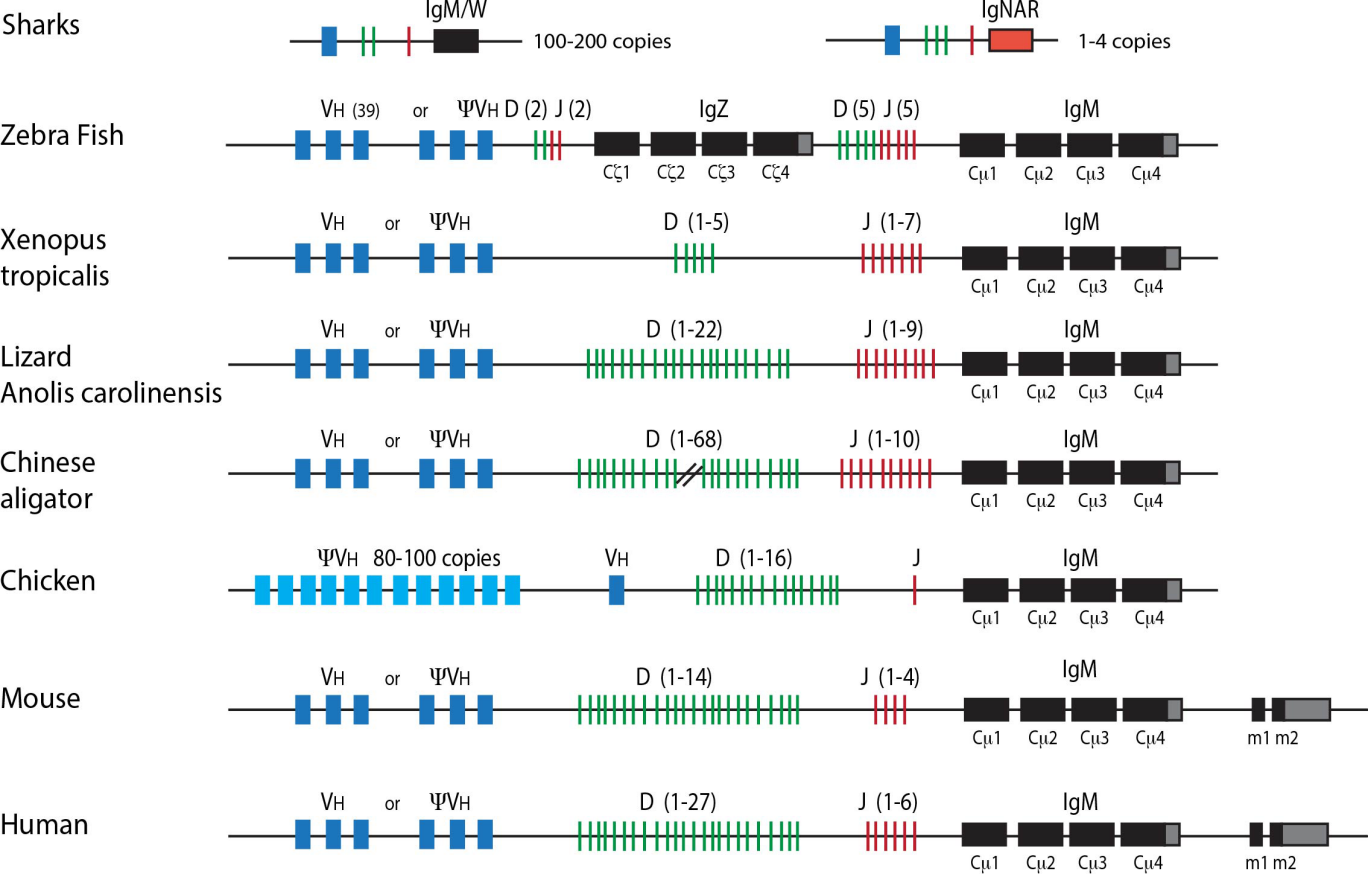
Gene segments forming the functional variable region in different jawed vertebrates. The gene segments are color-coded, with the V segments in dark blue, the pseudo-V segments in chicken in light blue, the D segments in green, and the J segments in red. The number of V, D, and J segments can vary significantly between species. The figure is made in Adobe Illustrator.

In mammals, the RAG-mediated recombination involving a large number of V, D, and J segments is the variability-generating mechanism. However, in chicken, the primary variability is instead generated by gene conversion. Chicken initially uses RAGs to assemble a functional transcription unit of the immunoglobulin heavy and light chains. However, almost all the variability is generated by gene conversion, using segments from 80 to 100 pseudogenes located upstream of this single rearranged gene (**Figure 11**) (149). The initial rearrangement involves a single V, one out of 16 D segments, and one single J segment (**Figure 11**). Following this initial rearrangement, short fragments from the different pseudogenes are inserted into the already rearranged V gene to diversify the antigen specificity, step by step, by a series of gene conversion events (149). This shows that very different mechanisms are used to generate variability by different animal groups, and the only thing that matters is that variability is being generated and ultimately that the individual survives to contribute to a new generation.

### 5.7 T-cell receptors

**Figure 12:**
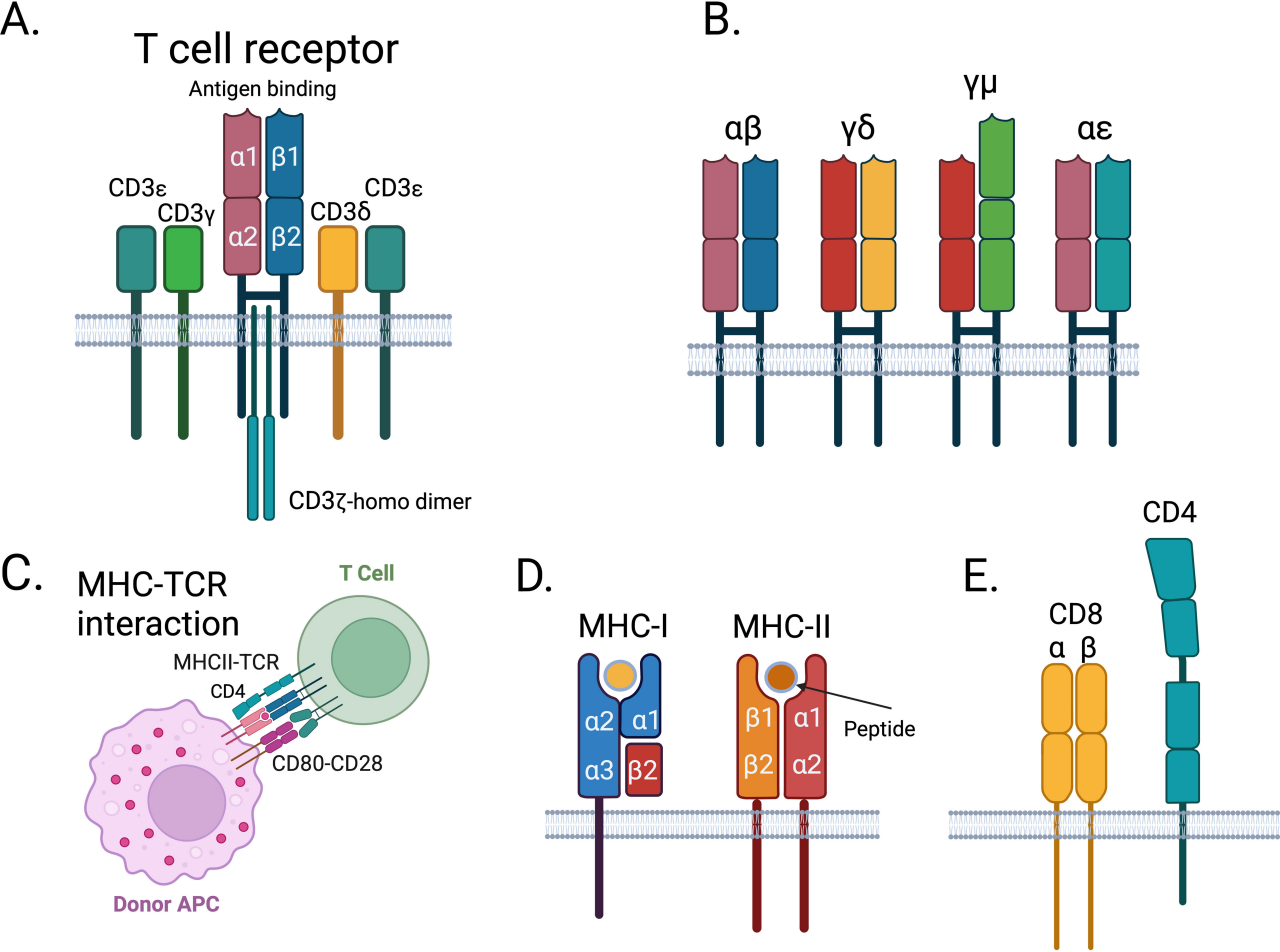
Schematic structures of the T-cell receptor, major histocompatibility complex (MHC), CD4, and CD8 proteins. The immunoglobulin-like domains are depicted as rectangles with rounded edges, whereas the antigen-binding regions in the T-cell receptors in **(A, B)** are shown as rectangles with a jagged top. The peptide-binding domains of MHC class I and II are in **(D)**, marked with α1 and α2 for class I and β1 and α1 in class II. In **(C)**, a schematic presentation of the interaction between a T cell and an antigen-presenting cell is depicted, illustrating the interacting molecules on their surfaces, including MHC class II, the T-cell receptor (TCR), CD4, and the CD80 interaction with CD28. **(E)** shows schematic structures of CD4 and CD8. The figure is made in BioRender.

T cells use TCRs for specific antigen recognition. Similar to antibodies, TCRs consist of immunoglobulin domain-containing molecules with two antigen-binding polypeptides, the conventional α chain and the β chain, or, in an alternative variant, the γ and δ chains, as present in a subpopulation of T cells. These four chains are all membrane-bound and have two extracellular domains, where the first, the outer, forms the variable antigen-binding domain (**Figure 12A**). The inner, which is located close to the membrane, shows no or very limited diversity and is primarily stabilizing the molecule and extending the molecule out from the surface of the T cell (**Figure 12**). The two chains contain a membrane-spanning region of approximately 20 amino acids. The variable domains of the TCR can connect with the antigen-presenting molecule, the MHC molecule (described later), on an antigen-presenting cell or a target cell. On the surface of the T cell, TCRs are in a complex with several additional non-varying proteins, known as the CD3 components (**Figure 12A**). These membrane-anchored accessory proteins are essential for signaling into the cell when the receptor has recognized its antigen. When the receptors are cross-linked, the intracellular region of the CD3 molecules becomes phosphorylated and connects to the cytoplasmic signaling molecules, and an intracellular signaling cascade is initiated. The αβ TCR is found in all jawed vertebrates but not in invertebrates or jawless fishes. The γδ TCR is also found in almost all jawed vertebrates, except for a large group of reptiles, including the majority of lizards and snakes, the squamate reptiles, which have lost both the γ and δ loci (150). Potentially as a compensatory mechanism, a part of the β locus has been duplicated, forming an α-chain binding partner. This new locus has been named the ε locus, and the protein product has been named the ε chain (**Figure 12B**) (151). One additional unconventional TCR chain has also been found in monotremes and marsupials, named the μ chain (152). This chain is longer and has an extra domain, and the V region is more similar to the heavy-chain V regions of immunoglobulins than other TCR V regions and, in particular, to the camelid single heavy-chain V regions that lack light chains (**Figure 12B**) (152). This μ chain seems to have evolved from the TCR δ locus (152). The binding partner of this chain is not known. The μ chain locus seems to have appeared during early mammalian evolution but has been lost in eutherian (placental) mammals (152).

### 5.8 MHC and antigen presentation

TCRs cannot recognize native antigens, which are the primary targets for immunoglobulins. To be able to recognize antigens, T cells need the antigen to be presented as peptides on MHC class I or II proteins. MHC class I molecules consist of an alpha chain with two antigen-binding domains and one membrane-proximal Ig domain (**Figures 12C**, **D**). A beta chain, named beta-2 microglobulin, without a membrane anchor, is then bound to the alpha chain to stabilize the molecule. The main variability of the MHC class I molecule is found in the alpha chain and here almost exclusively in domains 1 and 2, which are in direct contact with the antigen peptide (**Figures 12C**, **D**). MHC class II molecules have a very similar overall structure but do instead have two similar-looking chains, the alpha and beta chains, where both have one antigen-binding domain and one membrane-proximal Ig domain, and where both have transmembrane regions and a short cytoplasmic tail (**Figures 12C**, **D**).

Except for the highly variable immunoglobulins and T-cell receptors, the MHC molecules are the most highly variable molecules between individuals in the human genome. This is to ensure recognition of a maximal number of peptides from potential pathogens to be able to respond by the adaptive immune system. Almost all components of the antigen presentation machinery are found in one locus, the MHC. This includes the alpha chain of the MHC class I molecule, both the alpha and beta chains of the MHC class II molecules, the peptide transporters TAP1 and TAP2, proteases needed for peptide generation, and molecules needed for facilitating peptide loading on class II molecules, including the invariant chain and the MHC-DO and MHC-DM molecules. The beta-2 microglobulin, which is bound to the class I alpha chain and stabilizes the class I molecule, is in the human genome, located on another chromosome. However, when looking at the evolution of the MHC locus, evidence from cartilaginous fishes shows that the gene for beta-2 microglobulin was originally located in the MHC locus and has secondarily been moved to another genomic location (153). Sharks have all the components found in the human locus except for the DO and DM, which seem to have been added later. DO and DM seem first to be found in tetrapods (154). In sharks, DM and DO are potentially being replaced by alternative molecules involved in peptide loading of MHC class II, such as the DE group (154). The MHC locus also contains additional immune genes that have been named class III genes, including several complement components such as C4, C2, and factor B, but also the cytokines TNF-alpha and lymphotoxin. The majority of these genes are also present in the cartilaginous fishes, indicating a very early presence in the original MHC locus (153). An ancestral protovertebrate locus seems to have been the origin of the MHC locus as well as loci encoding Ig and TCR genes. This ancestral locus contains a complement C3-like molecule, an immunoglobulin V and C region-like gene, and a few proteases similar to the proteasome protease components of the mammalian MHC locus (153). This locus may have become four loci by the two whole-genome duplications that occurred during early vertebrate evolution (153). The V- and C-related gene fragments may then have undergone numerous gene duplication events and, by mutations and rearrangements, have been the origin of present-day MHC, immunoglobulin, and TCR genes (153). However, the different steps in this process are still, to a major extent, unknown.

### 5.9 CD4 and CD8

In order to stabilize the interaction between the MHC molecule and the TCR, two different cell surface proteins are essential: the CD4 and CD8 molecules (**Figure 12E**). The CD4 molecule binds to MHC class II and is therefore essential for T helper cells that interact with MHC class II on antigen-presenting cells. CD8 has the same function for cytotoxic T cells, by interacting with MHC class I molecules, where CD8 binds to MHC class I and stabilizes the interaction between a virus-infected cell and the T cell. CD8 is found both as a homodimer of two CD8-alpha chains or, more commonly, on peripheral T cells as a heterodimer of an alpha and a beta chain (**Figure 12**) (155). Both the alpha and beta chains are already found in cartilaginous fishes (156).

### 5.10 The CD28/CTLA4/ICOS/PD1 family of cell surface proteins

There are also several regulatory receptors on the surface of the T cell that interact with receptors on the antigen-presenting cell or the target cell. One important such family of receptors is the CD28/CTLA4/ICOS/PD1 family (**Figure 12C**) (157). Members of this family do not seem to be present in jawless fishes but appeared together with immunoglobulins and T-cell receptors with the emergence of jawed vertebrates. They also seem to have a very complex evolutionary history, including additional gene duplications and massive losses of members during the evolution of jawed vertebrates (157). CD28 is a potent costimulatory receptor on T cells, which is needed to avoid anergy induction in activated T cells (158). In contrast, CTLA4 is a negative regulator, primarily of activated T cells, to avoid overactivation by having a higher affinity for its ligand B7:1 and B7:2 on antigen-presenting cells and thereby blocking binding of CD28 by competition (158). ICOS is a costimulatory receptor that binds to its ligand ICOSL (159). PD1 is instead a negative regulator that, similar to CTLA4, primarily acts on activated T cells to dampen activation (158).

## 6 Adaptive immunity in jawless fishes

One of the most fascinating discoveries in the analysis of immune evolution has been the identification of the VLRs in jawless fishes (160). Both hagfish and lamprey have molecules that show overall structural similarity to those of TLRs and other LRR-containing molecules (161). These VLRs form the basis for an adaptive immunity of a complexity similar to the diversity of Igs and TCRs of jawed vertebrates. This is one of the most beautiful examples of convergent evolution. Proteins of completely different origin have, by convergent evolution, obtained almost identical biological functions.

**Figure 13:**
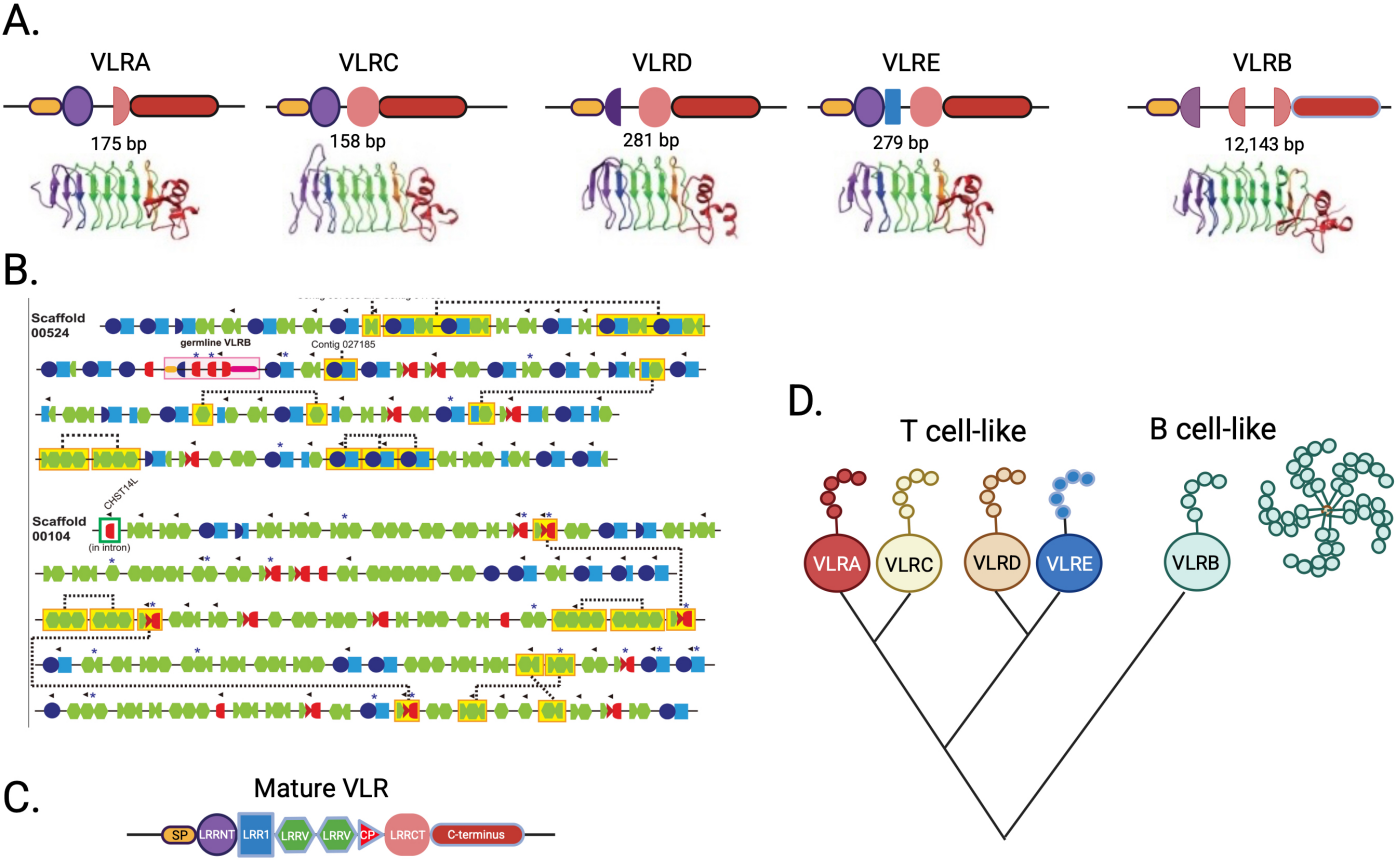
Variable lymphocyte receptors of lamprey and hagfish. Figure modified from figures in Das et al. (160, 163). **(A)** shows the unrearranged germline configuration of the five different variable lymphocyte receptor (VLR) loci presently identified. Below the gene structure is a schematic presentation of the 3D structure of the corresponding rearranged functional VLR proteins containing the internal LRRV repeats in green. The unrearranged gene does not contain the coding regions for these green parts of the protein; they are inserted by gene conversion to generate the functional final expressed molecule. The unrearranged gene contains the coding regions for the N- and C-terminal non-varying parts of the final VLR protein, shown as a yellow oval, a purple oval, a pink half or whole oval, and a red long oval. **(B)** shows two sequenced regions from the lamprey genome containing a large number of LRRV repeats. These LRRV regions are the gene segments forming the green antigen-binding part of the final VLR. **(C)** shows a fully rearranged mature VLR gene. **(D)** shows a phylogenetic tree of the five different VLR genes in the lamprey. The figure is made in BioRender.

Three types of VLRs were initially identified in both hagfish and lamprey: VLR-A, VLR-B, and VLR-C (162). VLR-B is the counterpart of IgM in jawed vertebrates and can be present on the surface of B cell-like cells to act as an antigen receptor and be secreted as a pentamer similar to IgM of jawed vertebrates (**Figure 13A**). In contrast, VLR-A and C function as T-cell receptors. VLR-A has been found to most closely resemble αβ TCRs of mammals, whereas VLR-C seems to be the counterpart of γδ TCRs (160). The process of variability generation is also different between jawless and jawed vertebrates. Jawed vertebrates use the recombination-activating genes RAG-1 and RAG-2 to combine the variable diversity and joining segments into one single open reading frame encoding the variable region of the immunoglobulin heavy chain, the light chain, and the TCRs. In contrast, the jawless vertebrates use a different system involving gene conversion-like mechanisms mediated by cytosine deaminases CDA1 and CDA2 to assemble the different leucine-rich repeats of the VLRs (160). CDA1 and CDA2 are members of the activation-induced cytidine deaminase family (AID/APOBEC), which is involved in somatic hypermutation and immunoglobulin class-switch recombination in mammals, as described earlier in this article (160).

Similar to Igs and TCRs, the VLRs are assembled from gene fragments located at separate positions in the genome (**Figure 13B**). The unrearranged VLRs do not contain the internal variable leucine-rich repeat (LRRV) sequences; they are located upstream and downstream of the core gene. By gene conversion, several of these LRRV sequences are introduced into this core gene to form the final functional VLR protein, where the antigen binding is confined almost exclusively to their central LRRV sequences (**Figure 13C**).

Very recently, two additional closely related VLR loci have been identified and named VLRD and VLRE. These two are more closely related to the VLRA and C than to VLRB and use at least partly the same V genes as VLRA and C (**Figure 13D**) (163).

In addition to lymphocytes and immunoglobulin and T-cell receptor-like molecules, several other similarities between the immune system in jawed and jawless vertebrates have been found, including several of the regulatory cytokines, such as IL-17, IL-1, and TNF family members, that have been identified based on their conserved primary sequence motifs in the lamprey (162, 164).

## 7 Cytokines, chemokines, and other soluble messengers of cell communication

Cytokines and chemokines play important functions in cell communication, during differentiation, as activators of cell proliferation, in guiding immune cells into specific regions of the lymph node, the spleen, and also into inflamed tissues. Cytokines are often pleiotropic by having multiple effects, sometimes even opposing effects, on different cell types. In the human genome, there are 41 different interleukins, several colony-stimulating factors, several tumor necrosis factors, several interferons, and a few related cytokines, such as LIF, TSLP, EPO, and TPO (164). In addition, there are almost 50 different chemokines, which are primarily involved in cell migration. A few of these cytokines and chemokines are relatively highly conserved and found also in lamprey and invertebrates, such as the cytokines of the IL-17 family, which are found in cnidarians and have expanded extensively in marine molluscs and echinoderms, but are not present in Porifera (165). For the majority of cytokines, the primary sequence conservation is generally relatively low (164). This is in contrast to their 3D structure, which is more conserved (164). A higher degree of primary sequence conservation has also been observed for their receptors, which can give clues to the presence of related cytokines and chemokines also in early chordates and invertebrates (164). The proteins involved in cell signaling from these receptors, including the JAK/STAT pathways, are also highly conserved (125). A detailed analysis of the lamprey genome has shown that almost all the different cytokine receptors found in jawed vertebrates are also found in the lamprey. It is only the erythropoietin EPO and IL-2/IL-15 receptors that seem to be absent, indicating that these cytokines may also be absent in lamprey and hagfish (164). A few extra copies of some receptors were also found in lamprey, which are not present in jawed vertebrates (164). The presence of these receptors indicates that the corresponding cytokines are there, although difficult to find due to high primary sequence divergence. The separation of the IL-2 and IL-15 alpha chains and the separation of IL-4, IL-21, and IL-13 seem to have occurred during jawed vertebrate evolution, in both cases by gene duplications, possibly to fine-tune the regulation of Th1 and Th2 responses in jawed vertebrates (166). Several of the receptors and the JAK/STAT pathways have homologs in *Drosophila* and even in Cnidaria, such as the IL-17 family described above, indicating a very early appearance of some of these receptors and their ligands (125). Several proteins with cytokine-like activities have been identified in *Drosophila*, including UPD1, UPD2, UPD3, Spätzle, Eiger, Pvf1, Pvf2, Pvf3, and the growth-blocking peptide (GBP) (92). The presence of cytokines as early as Cnidaria is maybe not surprising, as the possibility for cells to communicate is important for all multicellular organisms and particularly important for immune cells that are highly mobile and need to quickly expand or decrease in numbers upon infection or after the resolution of an infection.

## 8 Adaptive immunity in invertebrates?

Molecules with high variability have been found in a number of invertebrate species. However, the variability is, in general, many orders of magnitude lower than what is seen in vertebrates. One such example is the Down syndrome cell adhesion molecule (DSCAM) of *Drosophila*. Differential splicing has been estimated to generate 38, 016 different variants of this molecule (167). Initial studies have indicated that these molecules could act both as cell surface-bound antigen receptors and as soluble opsonins (167). However, later studies have challenged this view, and there is still no good proof that these molecules are involved in immunological memory similar to what has been observed for both the VLRs of jawless fishes, the Igs and TCRs for jawed vertebrates, and the CRISPR–Cas system of bacteria (168). Several findings point instead to a role for DSCAM primarily in communication between brain cells in both vertebrates and *Drosophila* (169). Highly variable molecules have also been found in other invertebrates, including the fibrinogen-related proteins (FREPs) of snails, the SP proteins of sea urchins, and the variable region-containing chitin-binding proteins (VCBPs) of the lancelet (170–174). Some highly variable molecules have also been identified in plants, including the leucine-rich repeat-containing molecules of plants, with similarities in structure to the TLR and the VLRs (175). These are involved in many different processes in the plant, including stress response, innate immunity, and development. However, their role in innate immunity is more like that of PRRs of vertebrates, such as TLRs and NLRs (175). The different variable molecules described above may potentially be involved in immunity by enhancing phagocytosis. However, they have low complexity and are involved in other major physiological processes, and clear evidence for a major role in immunity for most of these molecules is lacking, which has resulted in doubts about whether adaptive immunity has any role in immunity outside of vertebrates. Interestingly, for our view of the appearance of an adaptive immune system in chordates, genes for VLR-like proteins have been found in the lancelet, indicating that VLRs appeared early in chordate evolution. However, if they are part of an adaptive immune system, such as in lamprey and hagfish, is to our knowledge not yet known (176). Analysis of the genomes of amphioxus, the urochordate *Ciona intestinalis*, and the sea urchin has shown that massive expansions of other gene families have occurred, which most likely, at least partly, can compensate for the lack of a complex adaptive immune system. In the amphioxus, 72 TLR-related genes, 118 NLR-related genes, and 1, 200 C-type lectin genes have been identified (174). As mentioned earlier, a massive expansion of TLR genes has occurred in the sea urchin. The sea urchin also has 203 NLR-related genes and as many as 218 genes for scavenger receptor cysteine-rich (SRCR) domain (174). The presence of the variable molecules, such as the SP proteins in sea urchins and the FREP molecules of molluscs, does, however, indicate that evolutionary processes are ongoing to generate the first building blocks of a functional adaptive immune system in these invertebrate lineages.

## 9 Immunity in plants

In contrast to mice and humans, comparatively little has so far been performed on immunity in plants. However, the search for more pathogen-resistant variants of plants has boosted the screening for genes of interest and thereby increased our understanding of their immune system. The majority of genes identified during such screening efforts have been genes involved in extracellular and intracellular sensors of pathogen-related molecules, similar to the classical PRRs, the TLRs, NOD, and RIG receptors. Plants lack adaptive immunity and therefore have to depend entirely on innate immunity. They do not have migratory cells, such as macrophages, that can patrol tissues for the presence of pathogens to be removed. Instead, plants have developed very complex networks of PRRs to detect the presence of invading pathogens and then to activate several types of defense mechanisms. These mechanisms include the production of oxygen radicals, the initiation of apoptosis of infected cells, the triggering of cell wall thickening, the production of antimicrobial peptides, and the production of pathogen-targeting enzymes such as lysozyme against bacterial cell walls and chitinases against fungi and insect exoskeletons (50). Some molecules related to these mechanisms are constitutively produced, and others are only activated in response to a pathogen encounter, such as oxygen radicals and molecules participating in apoptosis, which would otherwise be very harmful for the plant if not tightly controlled. As mentioned above, plants have a very complex set of PRRs, which are either surface-located receptor-like kinases (RLKs) or receptor-like proteins (RLPs), where various ligand-binding exodomains recognize the PAMPs or DAMPs (177, 178). A large number of such PRRs have been characterized in different plant species where the ligands have been characterized and where the majority of them belong to leucine-rich repeat (LRR)-containing RLKs or RLPs (179). Ligands such as different flagellin epitopes, Ef-Tu, LPS, cold shock protein, peptidoglycan, and chitin epitopes have been identified as key targets for these LRR receptors and for other non-LRR-containing RLKs and RLPs (179). These non-LRR-containing molecules often belong to the intracellular nucleotide-binding class (NB) of PRRs (179). A strong response by these plant PRRs is often accompanied by localized cell death called the hypersensitive response, which can result in the termination of pathogen growth (179, 180). The direct response to PRR ligand recognition involves a rapid increase in cytosolic Ca^2+^ concentrations, extracellular alkalinization, and also often the production of reactive oxygen species, such as superoxide anions (O_2_^−^) and hydrogen peroxide (H_2_O_2_), and of transcriptional reprogramming (179).

In contrast to the majority of other effector mechanisms of plant immunity, a relatively detailed analysis of the large repertoire of antibacterial and antifungal peptides has been performed in plants. These antimicrobial peptides are in plants almost exclusively stabilized by cysteine bridges, with all from one to six such disulfide bridges (50). Primary, secondary, and tertiary structures also vary greatly between these peptides (50). Their functions also differ greatly and appear to be multifaceted. This is possibly to compensate for fewer other immune-related mechanisms. However, it is presently too early to draw any such conclusion before a broader analysis of the different immune functions and components in plants has been performed.

A major danger for plants is also insects, which feed on plants. Plants, therefore, need to find ways to counteract attacks by insects. Plants have lysozyme for protection against bacteria, similar to vertebrates and most multicellular organisms, but plants also have chitinases and 1, 3-beta-glucanases. Chitinases are enzymes that degrade chitin, a carbohydrate component of fungal cell walls and insect exoskeletons (181). Chitinases, together with 1, 3-beta-glucanases, are important in the defense against fungal infections (181). Plants also produce several toxic compounds upon insect or insect larval feeding on leaves and flowers, such as pyrethrins (182). They also signal to neighboring plants to start producing such compounds to be prepared for potential attacks by herbivores and pathogens by the production of volatile organic compounds (182).

It can also be mentioned that the bacterial CRISPR–Cas9 system has been used extensively as a tool in gene editing in plants to improve plant immunity (183, 184).

## 10 Summary

Immunity is essential for all living organisms. However, the systems used to obtain sufficient protection vary greatly. Some systems, such as most of the innate mechanisms, are old and present in most multicellular organisms and even in some single-celled organisms. In sharp contrast, it is questionable if many invertebrate species even have adaptive immunity and if they instead almost entirely depend on innate defense mechanisms. This indicates that adaptive immunity developed later during evolution and also independently in different animal groups. Very interesting is also the appearance of an almost complete set of components for the adaptive immune system of jawed vertebrates with the emergence of cartilaginous fishes, including antibodies, T-cell receptors, MHC molecules, and regulatory molecules, including B7 molecules and CD28-, CTLA-4-, and PD-1-related molecules. The parts of adaptive immunity that seem to have appeared somewhat later are 1) the isotype switch, which appeared with tetrapods; 2) the specific Fc receptors for IgA, IgG, and IgE, which appear with mammals; 3) the other members of the Fc receptor family, which are present in bony fishes but seems lacking in cartilaginous fishes; and 4) some components of the antigen presentation machinery, such as HLA-DO and DM, which seem to have appeared with tetrapods.

The seemingly rapid appearance of the basic components of the Ig- and TCR-based adaptive immune system from what appears as nowhere shows similarities to one of the most intriguing events in the history of life on Earth, the Cambrian explosion. In this event, most of the animal groups of today seem to have appeared from nowhere at the beginning of the Cambrian, between 541 and 510 million years ago (146). The jawless fishes use a completely different system for adaptive immunity and lack all of the central components of the adaptive immune system of jawed vertebrates. The origin of the many different components of the adaptive immune system of the jawed vertebrates is, therefore, somewhat of a mystery. Massive gene duplications, including two whole-genome duplications, may have been a contributing factor to the complex immune system we see in jawed vertebrates. A single V region and a single C region are present in the original locus that later became the MHC locus of jawed vertebrates, which is present in early chordates.

Interestingly, the VLRs of jawless fishes seem to have been the original system in early chordates, as related genes are found in amphioxus. With the emergence of jawed vertebrates, this system was apparently abandoned, and a completely new system was adopted, comprising essentially all existing components of today, which have been maintained and refined over the subsequent 450 to 500 million years of evolution in jawed vertebrates. The intriguing question is why this happened when the system in hagfish and lamprey, with their VLRs, seems to function so well and has variability in the same range as the human Ig-, TCR-, and MHC-based adaptive immunity. One likely explanation is that the VLR system was not at all developed when jawless and jawed vertebrates separated in evolution and that the systems have obtained their full potential in parallel. A possibility is therefore that jawed vertebrates did not abandon a functional system, as the fully functional VLR system may have developed hundreds of millions of years later.

An important and unresolved question is how the innate immune systems of invertebrates have diversified to perform functions that, in jawed vertebrates, are distributed between innate and adaptive immunity. Addressing this issue is central to a deeper understanding of immune system evolution in multicellular organisms. One long−standing hypothesis is that increased body size and elevated body temperature acted as major driving forces for the emergence of immunoglobulin− and TCR-based adaptive immunity. However, available evidence suggests that the earliest species in which this system evolved may have been relatively small animals inhabiting cold aquatic environments, challenging this view. Thus, while substantial progress has been made in identifying key events in immune evolution, many fundamental and intriguing questions remain unresolved.

**Figure 14:**
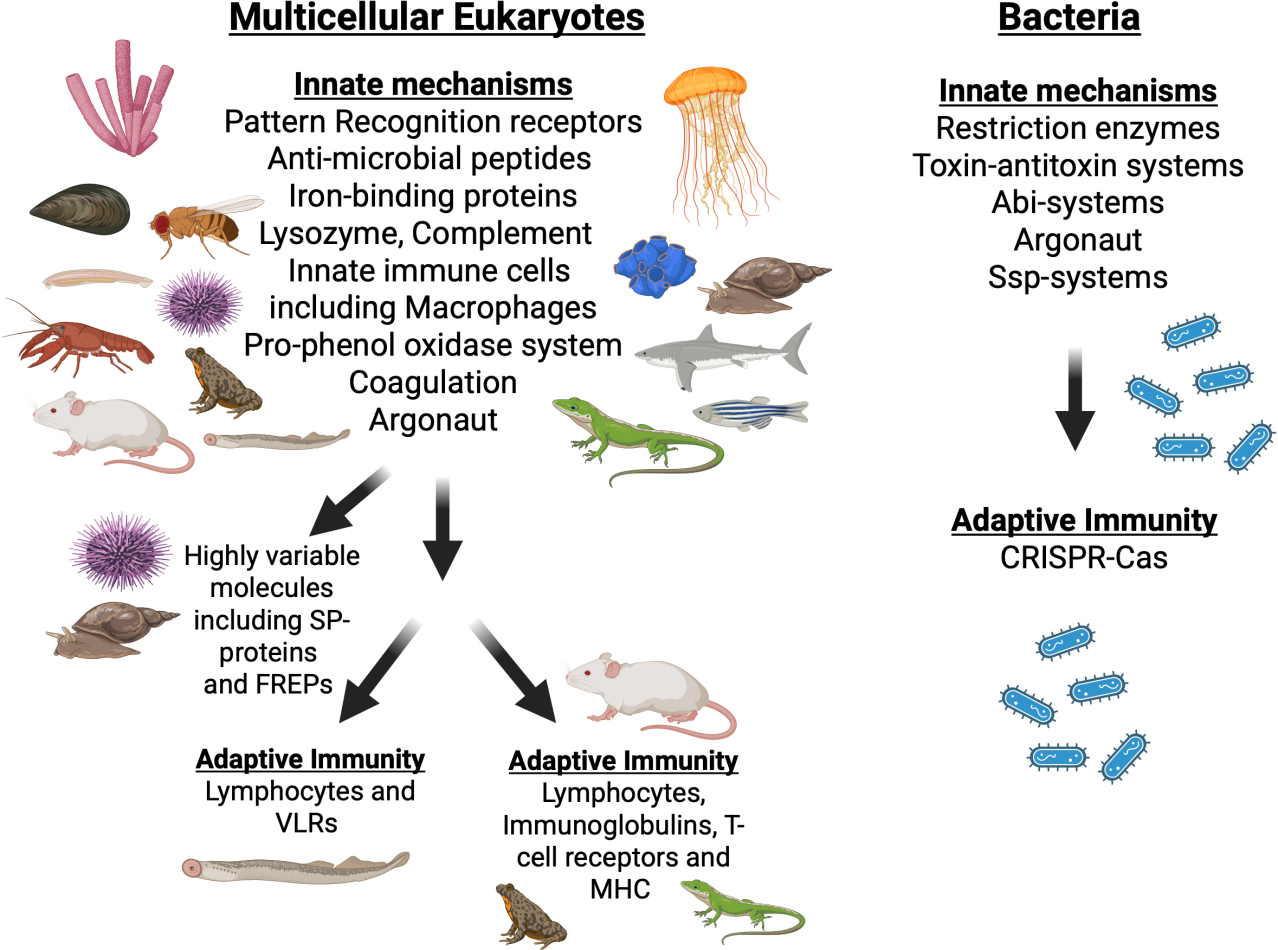
Immune mechanisms in multicellular eukaryotes and bacteria. The figure shows the major immune mechanisms of multicellular eukaryotes and bacteria involving both innate and adaptive mechanisms.

A summary figure of the various immune mechanisms used by different multicellular eukaryotes and bacteria is presented in **Figure 14**.

## 11 Concluding remarks

Evolution of immunity seems to have undergone several stepwise transitions and often in parallel in different organisms. Powerful innate immunity in bacteria and multicellular organisms has developed in parallel to sense PAMPs or self-related damage or stress-related DAMPs. Bacteria use the presence or absence of modified restriction sites, or the presence of saved pathogen-specific sequences as sensing systems, whereas multicellular organisms use Toll-like, NOD-like, and RIG-like receptors or similar PRRs to detect foreign substances or stress-related self-structures. Also interesting is the fact that very potent adaptive immune mechanisms have developed in parallel in bacteria and jawless and jawed vertebrates using completely different molecular mechanisms and proteins. Systems with considerably lower complexity have also been found in invertebrates, such as the FREPs in snails and SP proteins in sea urchins, where these systems indicate an ongoing process of evolution of a complex adaptive immune system also in invertebrates. The defense against infections is a constantly ongoing process of adaptation between the host and the infectious agent in a constant arms race.
